# Supplementary material for: HBM4EU Diisocyanates Study—Research Protocol for a Collaborative European Human Biological Monitoring Study on Occupational Exposure
Source: Int J Environ Res Public Health. 2022 Jul 20;19(14):8811. doi: 10.3390/ijerph19148811 (PMC9319997; doi:10.3390/ijerph19148811)
Supplement: Supplementary file 1 [file ijerph-19-08811-s001.zip › File S1 Standard Operating Procedures.pdf]

HORIZON2020 Programme
Contract No. 733032 HBM4EU

HBM4EU occupational biomonitoring study on diisocyanates

Standard Operating Procedures (SOPs)

WP8 - Targeted field work surveys and alignment at EU level

Published: January 2021

Entity	Name of person responsible	Short name institution	
Work Package Leader	Ovnair Sepai	DH	
Task leader	Tiina Santonen	FIOH	

Responsible author	Tiina Santonen	E-mail	tiina.santonen@ttl.fi
Short name of institution	FIOH	Phone	+358304742666
Co-authors	Karen Galea, Bernice Schaddelee-Scholten, Marika Loikala, Radia Bousoumah, Sophie Ndaw, Kate Jones, Elizabeth Leese, Jade Sumner, Henriqueta Louro, Maria João Silva, Susana Viegas, Lode Godderis, Jelle Verdonck, Katrien Poels, Simo Porras, Sirpa Hyttinen, Riitta Velin, Julia Catálan, Kukka Aimonen, Hannu Norppa, Sanni Uuksulainen, Thomas Göen, Glen McConnachie, Matthew Coldwell, Maurice van Dael, Radu-Corneliu Duca		

Table of Contents

Authors and Acknowledgements 3

1 Introduction 4

2 References 5

Annexes 6

Authors and Acknowledgements

Lead authors

Karen Galea (Institute of Occupational Medicine (IOM), United Kingdom);

Bernice Schaddelee-Scholten (Organization for Applied Scientific Research (TNO), the Netherlands);

Marika Loikala and Tiina Santonen (Finnish Institute of Occupational Health (FIOH), Finland);

Radia Bousoumah and Sophie Ndaw (French National Research and Safety Institute (INRS), France);

Kate Jones, Elizabeth Leese and Jade Sumner (Health & Safety Laboratory (HSL), United Kingdom);

Henriqueta Louro and Maria João Silva (National Institute of Health Dr. Ricardo Jorge (INSA), Lisbon, Portugal);

Susana Viegas (Escola Superior de Tecnologia da Saúde de Lisboa (ESTeSL), Instituto Politécnico de Lisboa, Portugal).

Contributors

Lode Godderis, Jelle Verdonck and Katrien Poels (Laboratory for Occupational and Environmental Health of the KU Leuven University, Belgium);

Simo Porras, Sirpa Hyttinen, Riitta Velin, Julia Catálan, Kukka Aimonen, Hannu Norppa and Sanni Uuksulainen (FIOH);

Thomas Göen (Institute and Outpatient Clinic of Occupational (IPASUM), Germany);

Glen McConnachie and Matthew Coldwell (HSL);

Maurice van Dael (Radboud Institute for Health Sciences (Radboudumc), the Netherlands);

Radu-Corneliu Duca (National Health Laboratory (LNS), Luxembourg).

(see annexes for detailed author lists)

This document has been created for the HBM4EU project. HBM4EU has received funding from the European Union’s Horizon 2020 research and innovation programme under grant agreement No 733032.

1 Introduction

Within the HBM4EU project, several priority chemicals were identified (https://www.hbm4eu.eu/the-substances/), which may be of concern for the European population. Several of those are also relevant at European workplaces, such as diisocyanates.

Diisocyanates are a group of chemicals containing two isocyanate functional groups (R–N=C=O). They are known to induce various health effects, including skin- and respiratory tract sensitization resulting in allergic dermatitis and asthma. There is also concern of potential genotoxicity and carcinogenicity of diisocyanates, with the degradation products and metabolites of 4,4' methylenediphenyl diisocyanate (MDI) and toluene diisocyanate diisocyanate (TDI) both being classified as Muta 2 and Carc 1B, with 2,4-TDA (toluenediamine) additional being classified as Repr. 2 (suspected of damaging fertility) and Skin Sens. 1. In addition, animal studies indicate the occurrence of lung tumours after exposure to both TDI and MDI (IARC 1999, ECHA, 2005, DECOS, 2018).

The two major diisocyanates in the European market are MDI (CAS 101-68-8) and TDI (CAS 584-84-9 for 2,4-TDI and CAS 26471-62-5 for the mixture of 2,4-TDI/2,6-TDI). A third diisocyanate with wide-spread use, especially in vehicle paints, is hexamethylene diisocyanate (HDI; CAS 822-06-0). In Europe, MDI, TDI and HDI account for more than 95% of the volume of diisocyanate production (ECHA 2017).

Since diisocyanates are widely used in very different applications (including manufacturing of polyurethanes (PU), hardeners in industrial paints, glues, varnishes and resins), occupational exposure during production and handling of these materials is a concern. Recently decided restriction on diisocyanates (Regulation (EU) 2020/1149) restricts the use of MDI, TDI and HDI in the EU unless specific conditions for workers' training and risk management measures (RMM) apply. In addition, there are plans to set an EU wide OEL for diisocyanates under Chemical Agents Directive (CAD).

Under HBM4EU a systematic review on the biomonitoring data on exposure to diisocyanates were performed (Scholten et al., 2020). The results show that about half of the studies published were prior to 2010 hence might not reflect current workplace exposure. There is also a large variability within and between studies and across sectors which could be potentially explained by several factors including worker or workplace variability, short half-lives of biomarkers, and differences in sampling strategies and analytical techniques. While several studies addressed exposure to TDI in flexible foam production, MDI in PUR production or HDI in paint spraying of vehicles in spray booths, less data was available on the occupational exposure to diisocyanates in the manufacturing of other vehicles such as aerospace, shipping and large commercial vehicles, construction sector where there are potentially several sources of exposure (e.g. sprayed insulation) and the use of MDI based glues and the manufacture of spray adhesives or coatings (Scholten et al., 2020).

Urinary diamines are the most commonly used marker to biomonitor exposure to diisocyanates. However, also new, more specific methods are available and may be worthwhile to develop further. This, together with above mentioned regulatory measures, support the selection of diisocyanates as the focus of the 2nd occupational study. The study can support the inclusion of diisocyanates into the Chemical Agents Directive (CAD) with an EU-wide OEL, which is currently under early discussion in the EU. The study can also provide data to support the evaluation of the effectiveness of the restriction of diisocyanates under REACH, which sets up a mandatory training system for the use diisocyanates at workplaces. Thus, in the case of diisocyanates, the study is likely to provide useful data for the implementation of EU policy, and the conduct of the targeted study seems only way how HBM4EU can address the policy questions identified for diisocyanates.

In the HMB4EU diisocyanate occupational exposure study (Santonen et al., 2020) exposed workers and controls from companies using diisocyanate-based products in the manufacturing and repair of large vehicles (non-booth spraying), in construction sector in e.g. insulation and flooring activities and in various sectors in the use of diisocyanate based hot-melt glues will be recruited from five countries, namely: Belgium, Finland, France, The Netherlands and United Kingdom (UK). In order to achieve comparable data in a harmonised way, the enclosed Standard Operating Procedures (SOPs) have been prepared. Every participating country is obliged to, as far as is reasonable possible, follow these procedures. The SOPs for the selection of participants and recruitment, information to the participants, informed consent (annex 1), completion of questionnaires (annex 2) and instructions for blood, urine, dermal, air, exhaled nitric oxide and buccal cells sampling (annexes 3-8), procedure for comparing occupational hygiene measurements with exposure estimates generated using REACH models (annex 9), and communication plan (annex 10), are annexed.

The general objective of the HMB4EU diisocyanate occupational study is to contribute to building a sound and sufficiently protective limit values for diisocyanate exposure at workplaces and to support the implementation and assessment of impact of up-coming REACH restriction on diisocyanates in EU. The study will also provide information on the reference levels of general population (data collected from controls) and provide new information on the applicability of different biomarkers in the assessment of diisocyanate exposure and risk at workplaces.

2 References

DECOS. (2018) Di- and triisocyanates. Health-based recommendation on occupational exposure limits (No. 2018/20). Health Council of the Netherlands. The Hague, The Netherlands. Available at https://www.healthcouncil.nl/latest/news/2018/11/28/recommendation-on-occupational-exposure-limit-isocyanates (last accessed 27th January 2021).

ECHA. (2005) European Union risk assessment report. Methylenediphenyl diisocyanate (MDI). CAS No: 26447-40-5. EINECS No: 247-714-0. Available at https://echa.europa.eu/documents/10162/9f8ad2fd-9b47-4eb6-9bf9-e0fc898f874d (last accessed 27th January 2021).

ECHA. (2017) Opinion on an Annex XV dossier proposing restrictions. Diisocyanates. ECHA/RAC/ RES-O-0000001412-86-174/F. Available at https://echa.europa.eu/documents/10162/737bceac-35c3-77fb-ba7a-0e417a81aa4a (last accessed 27th January 2021).

IARC. (1999) IARC monographs on the evaluation of carcinogenic risks to humans. Vol. 71. Available at https://monographs.iarc.fr/wp-content/uploads/2018/06/mono71.pdf (last accessed 27th January 2021).

Scholten B, Kenny L, Duca R-C, Pronk A, Santonen T, Galea KS, Loh M, Huumonen K, Sleeuwenhoek A, Creta M, Godderis L, Jones K. (2020) Biomonitoring for occupational exposure to diisocyanates: a systematic review. Annals of Work Exposures and Health; 64(6): 569-585. Available at https://doi.org/10.1093/annweh/wxaa038

Santonen T, Scheepers P, Schaddelee-Scholten B., Jones K, Galea, K, Viegas S, Bousoumah R, Ndaw S, Duca RC, Godderis L, Verdonck J, Pronk A, Louro H, Silva MJ, Martinsone I, Martinsone Z, Matisane L, Szigeti T, Sepai O. (2020) Detailed research plan for the occupational diisocyanate and E-waste study, Deliverable Report AD8.4, WP8 - Targeted field work surveys and alignment at EU level.

Annexes

Annex 1: Standard operation procedure for selection of participants and recruitment, information to the participants, informed consent (SOP 1)

Annex 2: Standard operating procedure for completion of company and worker questionnaires in diisocyanates study (SOP 2)

Annex 3: Standard operating procedure for blood sampling, including sample storage and transfer to be used in diisocyanates study (SOP 3)

Annex 4: Standard operating procedure for urine sampling, including sample storage and transfer to be used in diisocyanates study (SOP 4)

Annex 5: Standard operating procedure for assessing dermal exposure to diisocyanates (SOP 5)

Annex 6: Standard operating procedure for air sampling of diisocyanates (SOP 6)

Annex 7: Procedure for the collection of Exhaled Nitric Oxide (FeNO) samples (SOP 7)

Annex 8: Standard operating procedure for Buccal cells sampling, including sample storage and transfer (SOP 8)

Annex 9: Standard operating procedure for comparing occupational hygiene measurements with exposure estimates generated using the Advanced REACH Tool via the TREXMO model (SOP 9)

Annex 10: Communication plan for the occupational studies (diisocyanates and E-waste) (SOP 10)

Annex 1

SOP 1:
Standard operation procedure for selection of participants and recruitment, information to the participants, informed consent

WP 8
Task 8.5

Table of Contents

Authors and Acknowledgements 2

1 Introduction 2

2 Study design and participants 3

2.1 Target population 3

2.2 Sample size 3

3 Selection of sampling locations 3

4 Selection of participants, their recruitment and information 3

5 Informed Consent form 5

6 Assignment of participant and sample codes 5

7 References 6

Authors and Acknowledgements

Lead authors:

This document has been developed by Karen Galea (IOM, UK) and Bernice Schaddelee-Scholten (TNO, the Netherlands).

Contributions were received from Tiina Santonen (FIOH, Finland).

This SOP was developed with due consideration of the contents of the "SOP1: Standard operating procedure for selection of participants and recruitment, information to the participants, informed consent (SOP 1)" (lead authors Carina Ladeira, Edna Ribeiro, Susana Viegas, ESTeSL) developed for the HBM4EU occupational biomonitoring study on hexavalent chromium and other harmful chemicals (Porras et al., 2019).

This document has been created for the HBM4EU project. HBM4EU has received funding from the European Union's Horizon 2020 research and innovation programme under grant agreement No 733032.

1 Introduction

Standard Operation Procedure 1 (SOP 1) is focussing on the selection of participants and recruitment, information to the participants and informed consent.

2 Study design and participants

The study is focussed on small to medium sized enterprises (SMEs) using diisocyanates in specific industry sectors. The industrial sectors of interest include manufacturing and repair of large vehicles (non-booth spraying of e.g. boats/planes), the use of diisocyanate based hot-melt glues in different sectors, and construction sector, which includes different sources of diisocyanates exposure (floorings/screeds, insulation). Each participating country aims to collect samples from 50 exposed and 25 control subjects.

Participants will be recruited from five countries, namely: Belgium, Finland, France, The Netherlands and the United Kingdom (UK) during 2021.

2.1 Target population

The target population will be workers using diisocyanates in specific industry sectors. The industrial sectors of interest include manufacturing and repair of large vehicles (non-booth spraying of e.g. boats/planes), the use of diisocyanate based hot-melt glues in different sectors, and construction sector, which includes different sources of diisocyanates exposure (floorings/screeds, insulation). In addition, a control population of workers not involved in the above activities (most likely office staff of the same company) will also be recruited.

All adults with ages ranging from 18-70 years will be eligible. For some analyses, e.g., effect biomarkers characterization, specific inclusion/exclusion criteria may be defined because those biomarkers are affected by several confounding factors that should be reduced to avoid results misinterpretation.

2.2 Sample size

The target population size for this project is 50 exposed workers *per* country. Additionally, 25 workers (controls) *per* country will be also engaged in the study. Overall, this would result in 250 exposed and 125 control workers.

3 Selection of sampling locations

Sampling will be conducted in previously identified companies located in five countries, namely: Belgium, Finland, France, The Netherlands and the UK. Companies will be contacted and informed about the study aims and objectives. They will also be then invited to participate. The same approach will be followed for the workers' engagement in the study.

4 Selection of participants, their recruitment and information

Recruitment and information provision will be undertaken in the local language. For this purpose, a two-step approach will be undertaken, the first one for the company itself and the second one related to workers contact and information within the company. It should be noted that in some instances a site visit prior to the day of the actual sample collection may be necessary to obtain the necessary written consents, whereas in other instances the written consent may be obtained on the same day, immediately before commencement of the sample collections.

Company recruitment

Establishment of phone contact with the Company responsible. Upon company expressing interest in participating in the study, the information leaflet ***"INFORMATION FOR PARTICIPATING COMPANIES"*** will be issued. Where country specific rule requires its use (e.g., Belgium) upon a company agreeing to participate in the study an authorised representative of the Company must complete the ***"EMPLOYER CERTIFICATE OF INFORMED CONSENT"***. Where country specific rules do not require it's use completion of this certificate is not necessary. The company contact will then be requested to provide a list of names of those involved in activities that can implicate diisocyanate exposure, as well as a list of suitable control workers to approach.

Worker recruitment

Establishment of a contact with the worker, which is recommended to be done through a direct face-to-face meeting. The information leaflet on the study ***"INFORMATION FOR PARTICIPATING WORKERS"*** will be distributed and discussed during the first contact with the workers. Within this contact, a period to clarify all workers queries regarding the project is mandatory. Workers will be require to complete the ***"WORKER CERTIFICATE OF INFORMED CONSENT"*** if they are willing to give their informed consent to participate in the study. The same approach will be followed for controls.

Therefore, and following workers' acceptance to participate in the study, we will recruit workers in jobs that are likely to result in occupational exposure to diisocyanates. For example, workers involved in the manufacturing and repair of large vehicles (non-booth spraying of e.g. boats/planes), the use of diisocyanate based hot-melt glues in different sectors, and construction sector, which includes different sources of diisocyanates exposure (floorings/screeds, insulation).

In addition, we will recruit a group of unexposed (control) subjects, individually matched to the subjects for age (plus/minus 5-years), sex and smoking status (current smoker/ex-smoker/non-smokers). These control subjects will be selected from companies in the same geographical area with no known occupational exposure to diisocyanates or office staff from within the company where the worker participants are recruited.

Subjects should be in good health and present at work during the planned period of the study.

Blood samples to be used in in genotoxicity biomarkers study should only be obtained from workers that, in addition to the above criteria, should fulfil the following inclusion criteria: i) are under the age of 50; ii) are non-smokers or ex-smokers for more than six months; iii) have not been subjected to a medical exam such as a medical X-ray or Computerised Axial Tomography (CAT) scan in the last 3-months; iv) do not suffer or have suffered from cancer.

Information

Results communication should follow SOP 10 (Communication Plan). Moreover, the definition of which results, to whom and how the HBM results will be communicated should be defined in the beginning of the study following the General Data Protection Regulation requirements.

5 Informed Consent form

Where country specific rule requires its use (e.g., Belgium), the company should analyse and sign the Informed Consent Form ***(“EMPLOYER CERTIFICATE OF INFORMED CONSENT”***). The workers and controls accepting to participate in the study must sign the consent form (***“WORKER CERTIFICATE OF INFORMED CONSENT”***) before the collection of any information or samples. Workers are informed that after consent they are free to withdraw from the study at any time but that the research team will retain right to use any samples collected prior to the withdrawal in a confidential manner

The Informed Consent form can only be signed after receiving information explaining the aims of the study and all details required by the appropriate ethical regulations in each country. The researcher must be available for clarification during the reading and analysis of the consent form by the participants (workers and controls).

The Informed Consent forms should be archived and kept during all the study duration in each institution that participate the sample collection (not less than 5 years). Identification of companies and workers will not be used in all the process of sample handling and storage to guarantee the confidentiality needed.

6 Assignment of participant and sample codes

A standardised convention will be used to assign unique identification codes for all samples collected. The identification code convention is as follows:

Diisocyanate (D) - Country ID (XX) - Company ID (XX) - Participant ID (XXX) - Sample ID (AX/BX/LCX/RCX/BAX/EX/UX/WX)

‘D’ is to denote that the samples and data relate to the diisocyanate occupational study.

Country ID 'XX' is the country code, using the ISO Alpha-2 country codes for the participating countries (http://www.nationsonline.org/oneworld/country_code_list.htm).

Country	ISO Alpha-2 country codes
Belgium	BE
Finland	FI
France	FR
The Netherlands	NL
United Kingdom	UK

Company ID ‘XX’ is a two-digit running number of companies in each country (e.g. 01 for the first company recruited, 02 the second and so forth).

Participant ID 'XXX' is a three-digit running number of participants in each country (e.g. 001 for the first participant recruited, 002 the second and so forth).

Sample ID ‘AX/BX/LCX/…’ is one or two letters (A/B/LC/RC/BA/E/U/W) to identify the type of sample collected, followed by an one-digit identifier (X) to identify the running number of each type of sample for that worker (e.g. 1 for the first sample, 2 for the second and so forth). The letter code applied for the sample types is as follows:

Type of sample collected	Sample type code
Air	A
Blood	B
Buccal cells	LC (left cheek) or RC (right cheek)
Bulk sample for air samples	BA
Exhaled nitric oxide	E
Urine	U
Wipe	W

The following scenario is provided to illustrate the application of this convention.

A worker is recruited in United Kingdom. He is working in the first company recruited. He is the first worker recruited in that company and is providing his first two wipe samples. The sample identification codes assigned are therefore:

D-UK-01-001-W1

D-UK-01-001-W2

7 References

Porras S, Ladeira C, Ribeiro E, Viegas S, Uuksulainen S, Santonen T, Galea K, Cherrie J, Louro H, Ventura C, Silva MJ, Leese E, Jones K, Hanser O, Ndaw S, Robert A, Duca R-C, Poels K, Godderis L, Kiilunen M, Norppa H, Veijalainen H, Parshintsev E, Tuomi T, Ruggieri F, Alimonti A, Koch H, Bousoumah R, Antoine G, Jacoby N, Musgrove D. (2019) HBM4EU occupational biomonitoring study on hexavalent chromium and other harmful chemicals. Standard Operating Procedures (SOPs). WP8 - Targeted field work surveys and alignment at EU level. Available from URL: https://www.hbm4eu.eu/online-library/ (last accessed 27th January 2021)

Annex 2

SOP 2:

Standard operating procedure for

completion of company and worker questionnaires in diisocyanates study

WP 8

Task 8.5

Table of Contents

Authors and Acknowledgements.. 3

Summary 3

1. General introduction.. 3

2. Instructions to fill in the workplace questionnaire 4

2.1 Company and occupational health care information 4

2.2 Operational conditions 4

3. Instructions for completion of the workers post shift questionnaire (workers interviewed by researcher) 5

3.1 Background information about workers 6

3.2 Occupational history 9

4. Job description on different activities.. 10

5. Questionnaire on respiratory health and symptoms for workers (interview-led, filled also by control workers).. 11

6. Completion of questionnaire.. 12

7. Storing and reporting of gathered information.. 12

8. References 13

Appendix 1: Flash Card 1: Personal protective equipment used for jobs 1-4 14

Appendix 2: Flash Card 2: Personal protective equipment used for job 5 15

Appendix 3: Flash Card 3: Local exhaust ventilation used for job 5........................ 16

Authors and Acknowledgements

Lead authors:

This document has been developed by Marika Loikala and Tiina Santonen from the Finnish Institute of Occupational Health (FIOH), Finland.

Contributions were received from: Kate Jones (HSE, UK), Lode Godderis (KU Leuven, Belgium) and Simo Porras (FIOH).

This SOP was developed with due consideration of the contents of the "SOP2: Standard operating procedure for completion of company and worker questionnaires" (lead authors Sanni Uuksulainen (FIOH, Finland), Simo Porras (FIOH) and Karen Galea (IOM, UK)) developed for the HBM4EU occupational biomonitoring study on hexavalent chromium and other harmful chemicals (Porras et al., 2019).

This document has been created for the HBM4EU project. HBM4EU has received funding from the European Union's Horizon 2020 research and innovation programme under grant agreement No 733032.

Summary

This SOP defines:

- ✓ Instructions to fill in the workplace questionnaire
- ✓ Instructions for completion of the workers post-shift and controls questionnaire
- ✓ Instructions to provide job descriptions on different activities

The researcher should ensure that all questions are completed and that handwriting is legible. It is recommended that capital letters are used to record free text answers to aid in reading at the time of data entry.

1. General introduction

This SOP 2 – "Completion of company and worker questionnaires" is designed to support a targeted occupational study on isocyanate exposure performed under task 8.5.

This SOP has been created with the premise in mind that every participating country is obliged to try, as far as is reasonably practicable, to follow the HBM4EU documents to achieve comparable data in a (as much as possible) harmonised and consistent manner.

As it is stated in SOP1 (Selection of participants and recruitment, information to the participants, informed consent), the target population are workers using diisocyanates following industry sectors: manufacturing and repair of large vehicles (non-booth spraying of e.g. boats/planes), the use of diisocyanate based hot-melt glues in different sectors, and construction sector, which includes different sources of diisocyanates exposure (floorings/screeds, insulation). The exact processes used in the companies and by workers will be specified during the collection of contextual information, which is guided on this SOP.

Four questionnaires will be used to collect relevant contextual information for the study:

1. Self-completed company questionnaire
    - to be completed by the company representative, prior to the sampling campaign commencing
2. Interview led post-shift worker questionnaire
    - to be completed by the researcher while interviewing the worker, and
    - to be completed as close as possible to the end of work shift.
3. Job descriptions (interviewed by researcher during the sampling)
4. Interview led questionnaire on respiratory health and symptoms for workers

It should be noted that some questions will not be applicable to the control group (those participants not exposed to isocyanates) and these questions will be clearly identified as such.

2. Instructions to fill in the workplace questionnaire (administered by company representative)

Questionnaire A is to be completed by the company representative before the sample collection campaign starts. The questionnaire should all be self-explanatory. However, some further explanatory information is provided below should the company representative require further information. This explanatory information also acts as an aide memoir for the researcher.

Please ask the representative to fill in the questionnaire and return it directly to you (researcher) once completed.

Questionnaire explanatory text:

2.1 Company and occupational health care information

- Information on sector and description of the workplace are needed to be able to present the study results in aggregated form. Sector of use and nature of the business can be described in free text.
- The researcher will fill the NACE Rev.2 code. Copy corresponding NACE code (with 4 digits) and label text from the link. The classification is available in several EU-languages: http://ec.europa.eu/eurostat/ramon/nomenclatures/index.cfm?TargetUrl=LST_NOM_DTL&StrNom=NACE_REV2&StrLanguageCode=EN&IntPcKey=&StrLayoutCode=HIERARCHIC

  Most of the sectors of use in this study belong to classes 30 (Manufacture of other transport equipment), 41.2 (Construction of residential and non-residential buildings) 25.1 (Manufacture of structural metal products) and 35.3 (Steam and air conditioning supply).

2.2 Operational conditions

- The work tasks performed in the company need to be ticked, which will then direct the company representative to the sections that they need to complete. Sections of the questionnaire relating to work tasks not relevant to the company are omitted.
- The researcher should double check that the corresponding sections of the ticked work tasks are completed.

Section 1 - Operational conditions of Jobs 1-4:

1. Use of diisocyanate based glues, adhesives or sealants
2. Coating large surfaces (e.g. floors) with polyurethane coatings
3. Spray application of urethane foam in construction or boat manufacturing sector
4. Spray coating of vehicles with di-isocyanate containing paints and primers

- *Each section is to be completed if the company undertakes these work activities.*
- The specific isocyanate and quantity in the product used, consumption of the isocyanate containing product and size of the parts installed/sprayed/glued are asked because they affect the level of exposure.
- The number of employees working on these activities is asked in order to obtain a total number of workers who might be exposed to isocyanates.

Section 2 - Operational conditions of Job 5:

5. Welding, grinding and flame cutting of polyurethane painted materials or e.g. polyurethane-insulated heating pipes

- *This section is to be completed only if the company undertakes this work activity.*
- The frequency of this activity and the size of parts worked with are asked because they affect the level of exposure.
- The number of employees working on surface treatment activities is asked in order to get a total number of workers who might be exposed to isocyanates.

Section 3 - Previous measurements

- *This section is to be completed by all respondents*
- Background information of the previous exposure measurements is very important when estimating the risks of the exposure. If data on previous measurements is available for the researchers, the exposure trends can be determined. The researcher should highlight the confidential nature of providing the previous measurement data.
- If the company representative is unaware of the type of previous measurements that have been collected or the years in question, this should be recorded as free text on the questionnaire as 'Don't know'. A request should then be made for this information to be followed up and provided, where possible, by no later than the time of completion of the sampling campaign at the site.

Upon return of the questionnaire, the researcher should ensure that all questions are completed and that handwriting is legible. In the event that it is difficult to read the handwriting, the researcher should ask for clarification and rewrite the response in their own writing. The researcher is also required to complete the NACE Rev.2. Finally, for questions where a range of options relate to an answer e.g. grams or litres; daily, days/week or days/month, the researcher should double check with the respondent which option their response relates to.

3. Instructions for completion of the workers post shift questionnaire (workers interviewed by researcher)

Questionnaires B and C are interview-led with the responses being entered by the researcher. The interview-led questionnaire is to be completed as close as possible to the end of work shift where possible. The researcher can ask the site if the workers can finish a little earlier to allow the completion of the questionnaire. If this is not possible, the questionnaire should be filled in the next possible moment with due consideration of worker and researcher availability. The administration of the questionnaire should take place (where possible) in a quiet area, free from distractions.

Questionnaire is divided into two parts:

- Background information about worker
    - *To be completed for both exposed workers and controls.*

    - Personal habits (cigarette, alcohol consumption, diet) asked are relevant for data analysis.
- Occupational history
    - *To be completed for both exposed workers and controls.*
    - Please ask about all jobs lasting more than 12 months since leaving school or full-time education.

The interviewer should use capital letters to record free text answers to improve legible.

3.1 Background information about workers (to be completed for both exposed workers and control group)

- **Worker ID:**

A standardised convention will be used to assign unique identification codes for all workers and controls. The identification code convention is as follows:

Diisocyanate (D) - Country ID (XX) - Company ID (XX) - Participant ID (XXX)

'D' is to denote that the samples and data relate to the diisocyanate occupational study.

Country ID 'XX' is the country code, using the ISO Alpha-2 country codes for the participating countries (http://www.nationsonline.org/oneworld/country_code_list.htm).

Country	ISO Alpha-2 country codes
Belgium	BE
Finland	FI
France	FR
The Netherlands	NL
United Kingdom	UK

Company ID 'XX' is a two-digit running number of companies in each country (e.g. 01 for the first company recruited, 02 the second and so forth).

Participant ID 'XXX' is a three-digit running number of participants in each country (e.g. 001 for the first participant recruited, 002 the second and so forth).

The following scenario is provided to illustrate the application of this convention.

A worker is recruited in Finland. He is working in the first company recruited and he is the first worker recruited in that company. The worker identification code assigned is therefore: D-FI-01-001

- **Information related to the sample collection:**

Fill in the information regarding the samples collected. Pay attention to inform the actual sampling time for each sample. Note that not all samples may be collected. In which case "N/A" (not applicable) should be recorded in the relevant box.

A standardised convention will be used to assign unique identification codes for all samples collected. The identification code convention is as follows:

Diisocyanate (D) - Country ID (XX) - Company ID (XX) - Participant ID (XXX) - Sample ID (AX/BX/LCX/RCX/BAX/EX/UX/WX)

‘D’ is to denote that the samples and data relate to the diisocyanate occupational study. Country ID 'XX', Company ID ‘XX’ and Participant ID 'XXX' are as above.

Sample ID ‘AX/BX/LCX/…’ is one or two letters (A/B/LC/RC/BA/E/U/W) to identify the type of sample collected, followed by an one-digit identifier (X) to identify the running number of each type of sample for that worker (e.g. 1 for the first sample, 2 for the second and so forth). The letter code applied for the sample types is as follows:

**Type of sample collected**	**Sample type code**
Air	A
Blood	B
Buccal cells	LC (left cheek) or RC (right cheek)
Bulk sample for air samples	BA
Exhaled nitric oxide	E
Urine	U
Wipe	W

The following scenario is provided to illustrate the application of this convention.

A worker is recruited in Finland. He is working in the first company recruited. He is the first worker recruited in that company and is providing his first two wipe samples. The sample identification codes assigned are therefore:

D-FI-01-001-W1

D-FI-01-001-W2

- **Company name and department:** Company name is already known but name of department will need to be requested.

- **Worker name**: Name of the worker is needed in order to be able contact the worker in the future to tell him/her about his/her personal results or regarding the use of his/her stored sample(s) and personal data in the future studies. The name will be replaced with a code to protect worker's privacy.

- **Biological sex:** The background exposure to chemicals may differ in men and women). Circle response. If an individual advises, they do not wish to respond to this question, it should be left blank.

- **Date of birth:** This question is essential to identify potential differences in human exposures, as well as susceptibility associated with the age. Record as dd/mm/yyyy.

The front page of the worker questionnaire, containing the personal information, should be removed from the rest of the questionnaire. Before doing so please check that the remaining pages of the worker questionnaire have the relevant unique identification codes entered.

- **Height and current weight:** This information is used to calculate body mass index (BMI). Researcher to take care in ensuring correct units are assigned.

- **Free description of occupation:** Please describe as detailed as possible to help to choose the relevant ISCO-code (see the ISCO-08 classification in Appendix 2). Copy corresponding ISCO-08 code (with 4 digits) and text label from the link. The classification is available in English, German and French:
  http://ec.europa.eu/eurostat/ramon/nomenclatures/index.cfm?TargetUrl=LST_NOM_DTL&StrNom=CL_ISCO08&StrLanguageCode=EN&IntPcKey=&StrLayoutCode=HIERARCHIC
  Most of the occupations in this study belong to groups 71 and 72.

- **Outside or Inside work:** Exposure may vary depending whether the work is done outside or inside. Air flows or wind conditions may lower the exposure. Circle response

- **Duration of work shifts:** Please enter typical duration of a work shift with partial hours being recorded as follows: 30 mins is 0.5 hours; 15 mins is 0.25 hours. Therefore, a 7 and half hour work shift would be recorded as 7.5 hours.

- **Type of work shifts:** Note ‘back’ shift typically refers to a shift starting in the afternoon and finishing in the evening, e.g. 14.00-22.00

- **Home address** is needed in order to be able contact the worker in the future to tell him/her about his/her personal results or regarding the use of his/her stored sample(s) and personal data in the future studies. Worker's contact details will be stored exclusively for this purpose and will not be disclosed to any third party.

- **Location and related characteristics**: These questions aim to characterize the environment where the participant lives, as differences could exist in human exposure associated with the area of residence. Urban areas are very developed, meaning there is a density of human structures such as houses, commercial buildings, roads, bridges, and railways. "Urban area" can refer to towns, cities, and suburbs. In general, a "Rural area" is a geographic area that is located outside towns and cities. In other words, whatever is not urban is considered rural.
  Circle response

- **Industrial plants, incinerators or landfill sites in the surroundings of house:** It is necessary to collect information on facilities considered as potential sources of exposure to pollutants, which might lead to differences in human exposure levels. This question provides information on the general characteristics of the living environment (e.g. if the house is located in a heavily industrialized area there might be high background exposure). Background exposure may have an effect on nitrogen oxide (FeNO) concentration in exhalation air and also in regards of symptoms.
  Circle response. In the event that a Yes response is given prompt for the distance in km. In the event that the respondent indicates that they do not know the answer to this question ‘Don’t know’ should be recorded.

- **Vehicular traffic density and smoking habits (including all tobacco and e-cigarette products):** This information is relevant in regards of symptoms and these factors may also have an effect on nitrogen oxide (FeNO) concentration in exhalation air. Vehicular traffic density has meaning for effect markers too.
  The researcher should circle whether the respondent is a current smoker and if not, a former smoker. To assist the respondent in estimating the number of cigarettes smoked per day, a standard cigarette pack contains 20 cigarettes.

- **Consumption of other beverages, dietary habits, medical X-ray or computerised axial tomography (CAT), cancer and alcohol consumption:** These questions are relevant in regards of effect markers. If sample effect markers are not collected these questions can be deleted/dismissed.
  Other beverages refers to e.g. coffee, tea and energy drinks (sports drinks or gels, other caffinated drinks etc.). Circle which additional beverages are consumed and for those indicated how many times in a typical working day. Other dietary habits may include gluten free, lactose free diets and should be recorded as free text.
  If the workers responds 'yes' on a CAT or cancer questions their blood sample number Tube 1 will not be analysed for these biomarkers and a note of this should be made on the corresponding blood sample information form (see blood sampling SOP).
  When estimating alcohol consumption, it may help to ask, how many pints of beer/ glasses of wine/ glasses of spirit is consumed at a time. The researcer should highlight that the samples will not be analyzed for alcohol (nor for prescription or illegal drugs).

- **Recreational activities or hobbies:** Some recreational activities (e.g. machining of polyurethane containing products, home renovation and repair work including the use of adhesives, sealants, polyurethane foams, paints or coatings, motor vehicle repair including especially spray painting) may result in exposure to isocyanate. If the respondent indicates that they do have recreational activties that may cause additional isocyanate exposure, details of what they are should be recorded.

3.2 Occupational history (to be completed for both exposed workers and control group)

The information on occupational history and the exposure years are used when assessing the total cumulative exposure. Please, list all the possible work periods in the activities mentioned.

Researcher should start by asking about the respondents' current job (mentioning that this will be discussed in more detail if they are an exposed worker) and then ask the respondent to work back from this job through the jobs they have had. As a prompt it may be helpful to ask what year the respondent left school to ensure that all work periods are covered. In the event that the respondents work did not involve any of the activities of interest the activity boxes should be left unticked. In the event that the respondent did not work during a particular time period, for example, due to a period of study, unemployment or maternity leave this should be recorded as 'not employed' for the time period in question with the activity boxes left unticked.

Start and finish years should be recorded as YYYY, e.g. 1990.

Job description – The researcher should record which work task was performed by the participant which will then guide the next set of questions to be asked.

In the event that the respondent indicates that they did not complete any of these activities (in other words they are part of the control group), they should be advised that the questionnaire is complete and thanked for their contribution to the project.

4. Job description on different activities (interview-led, to be completed for only the exposed workers)

Questionnaire D is interviewed by the researcher during the sampling. *Control group participants do not need to respond to these questions.*

Job 1: Use of diisocyanate based glues, adhesives or sealants

Job 2: Coating large surfaces (e.g. floors) with polyurethane coatings

Job 3: Spray application of urethane foam in construction or boat manufacturing sector

Job 4: Spray coating of vehicles with di-isocyanate containing paints and primers

Job 5: Welding of polyurethane-insulated district heating pipes

After selection of the job the respondent will be asked only the questions relevant to that given job. All other questions will be left blank.

- Please select all type of work tasks the worker has been involved in today, not only the main task.

- Ask the requested contextual information:
    - *All jobs:* duration (hours / mins) and frequency of the tasks (times per week)
    - *Job 1:* Process type – record manual or automatic.
    - *All jobs:* Risk management by personal protection equipment (PPE). Show Flash Card 1 (see Appendix 1) to assist respondent in their response concerning PPE use and record the relevant numbers on the form. If ‘other’ RPE record ‘4’ and details of what was used. If other PPE is used, record ‘8’ and details of what was used.
    - *Jobs 1 – 4:* If local exhaust ventilation (LEV) was in use or not as this is very important in assessing adequate risk management of exposure. Record ‘yes’ or ‘no’
    - *Job 5:* Use of local exhaust ventilation (LEV) is very important in assessing adequate risk management of exposure. Show Flash Card 3 (Appendix 3) and record relevant number.
    - *Job 2:* Circle was the spreading done standing up or on hands and knees. This has an impact especially to exposure via skin.
    - *Job 4:* If spraying outside of spray booth, please circle was there spraying above the head height or not.

- It is important to know whether the respiratory protection equipment (RPE) (mask) has been fit tested. To assist the respondent fit testing can be explained as a method of checking that a tight-fitting facepiece matches the wearer’s facial features and seals adequately to their face. If they respond ‘Yes’, the year of testing should be recorded as 4-digits, e.g. 2017.

- Record as ‘yes’ or ‘no’ whether the worker has received information, instruction or training on the use of safe working practices for this activity.

- What kind of general ventilation is in the workplace? Tick the right answer.

- Hygiene facilities – tick all that apply. In the event that ‘other’ is indicated, further details should be recorded as free text.

- If the work conditions were not normal, please specify all the possible problems during the working day (e.g. problems with mask or extraction not working) as this may reflect to the level of exposure.

Operational conditions during welding

- Material used – circle whether this is ‘polyurethane foam’ or ‘other’. If ‘other’ is indicated ask what material was used and record free text response.
- Welding method used – tick all that were used during the sampling period. If ‘other’ is indicated, record free text response.
- If gas welding, e.g. TIG, then what gas mixture was used? If known, this should be recorded numerically as a percentage. If unknown enter ‘don’t know’
- Was the base metal painted (circle response: yes, no or don’t know)?
- Record the welding voltage and current numerically where known, otherwise circle ‘don’t know’.
- Where do you weld? This question relates to the locations where the participant welded during the sampling period. Tick all that apply and where confined spaces are indicated; ask respondent for an estimate of the size of the space in m^3.
- What kind of general ventilation is in the workplace? Tick the right answer and if mechanical please specify was both supply and exhaust air mechanical or only exhaust air.
- Record whether the welder needed to position his/her head in the fume plume (tick the box that applies)?

5. Questionnaire on respiratory health and symptoms for workers (interview-led, filled also by control workers)

Questionnaire E can be completed following the post-shift questionnaire. Also control workers should fill in this. The administration of the questionnaire should take place in a quiet area, free from distractions. Responses may be entered by the researcher or the worker. However, in the latter case the researcher should be near to answer to the questions workers may have.

The aim of the questionnaire is to find out whether exposed workers experience more respiratory symptoms than non-diisocyanate exposed controls.

1. **Medical problems:** these are important background information which may explain some symptoms experienced by the workers. Only those diseases diagnosed by the doctor are included. If the worker has any other respiratory disease (not listed in the table), this should be indicated. Heart diseases, like angina pectoris, may explain some of the symptoms listed below, e.g. shortness of breath in physical activity.

2. **Asthma**: If the worker has been diagnosed with asthma, it is useful to know how well it is currently controlled by the treatment. If rescue inhalators are often needed, it indicates poor control of the disease.

3. - 5. **Cough, wheezes, whistling and chest tightness, shortness of breath**: recurrent cough, wheezes, whistling and chest tightness, shortness of breath are common symptoms of asthma. These may wake you up in the night or be worse in the morning. It is important to find out whether

these symptoms are better on days off and holidays since this information may support work-relatedness of the symptoms. Transient cough related to common cold should not be taken into account here.

6. **Eye and nasal symptoms:** Sensitization to diisocyanates may also include nasal symptoms, like runny nose. In addition, also exposure to irritating substances at workplace may cause eye and nasal symptoms, which should be indicated. Symptoms clearly related to common cold should not be taken into account here.
7. This question is to find out whether the worker has sought medical attention for his/her symptoms. It can give indirect information on how severe the symptoms have been.
8. **COVID-19 Symptoms and diagnosis:** This information is collected since recent viral infection and vaccination will interfere in the inflammatory markers to be analysed.

6. Completion of questionnaire

Upon completion of the questionnaire, participants should be informed that the interview is now complete and thanked for their contribution to the project.

7. Storing and reporting of gathered information

The gathered hard copy questionnaires should be placed in secure storage which is accessible only to designated members of the project team.

The front page of the worker questionnaire, which contain the personal information, should be removed from the rest of the questionnaire. Before doing so check that the remaining pages of the worker questionnaire have the relevant unique identification codes entered. The first page of the worker questionnaire should be stored in a secure physical storage separate to the rest of the coded questionnaire so to ensure confidentiality of the collected information.

The company questionnaire should also be stored in a secure physical storage separate. The company questionnaire should remain intact, e.g. there is no need to remove the front page.

The hard copy questionnaire data should be entered into the central electronic template, that will be created once we have some results.

It is important that information entered into the hard copy questionnaires is recorded as per the instructions given in this SOP as this information will need to be entered into the data template for the overall diisocyanate study.

Care must be taken to follow the instructions accompanying the data template. The template must NOT be modified in any way - DO NOT add / remove columns, or alter the drop down lists, or merge cells. In the event that the template is modified or provided data does not follow the instructions, templates will be returned to the data provider for correction.

Both hard copy and electronic data should be archived and kept for 5 years, with storage of these files being in compliance with relevant national and European data protection legislation.

8. References

Porras S, Ladeira C, Ribeiro E, Viegas S, Uuksulainen S, Santonen T, Galea K, Cherrie J, Louro H, Ventura C, Silva MJ, Leese E, Jones K, Hanser O, Ndaw S, Robert A, Duca R-C, Poels K, Godderis L, Kiilunen M, Norppa H, Veijalainen H, Parshintsev E, Tuomi T, Ruggieri F, Alimonti A, Koch H, Bousoumah R, Antoine G, Jacoby N, Musgrove D. (2019) HBM4EU occupational biomonitoring study on hexavalent chromium and other harmful chemicals. Standard Operating Procedures (SOPs). WP8 - Targeted field work surveys and alignment at EU level. Available from URL: https://www.hbm4eu.eu/online-library/ (last accessed 27th January 2021).

Appendix 1: Flash Card 1: Personal protective equipment used for jobs 1-4

(use of diisocyanate based glues, adhesives or sealants; coating large surfaces with polyurethane coatings; spray application of urethane foam in construction or boat manufacturing sector; spray coating of vehicles with di-isocyanate containing paints and primers)

1. Powered or air-fed, filtering respirator

2. Reusable half or full-face mask respirator (not powered or air-fed)

3. Disposable face mask

4. Other Respiratory Protection Equipment (please specify)

5. Coveralls

6. Reusable Gloves

7. Disposable gloves

8. Other (please specify)

Appendix 2: Flash Card 2: Personal protective equipment used for job 5

(welding, grinding or flame cutting of polyurethane-painted materials or e.g. polyurethane-insulated heating pipes)

1. Welding helmet with powered or air-fed, filtering respirator
2. Welding helmet with half mask reusable dust respirator
3. Welding helmet with disposable particulate respirator
4. Welding helmet without any respirator
5. Welding helmet with other respiratory protection equipment (please specify)
6. Fire/flame resistant clothing
7. Welding gloves
8. Other gloves
9. Other (please specify)

Appendix 3: Flash Card 3: Local exhaust ventilation used for job 5

(welding, grinding or flame cutting of polyurethane-painted materials or e.g. polyurethane-insulated heating pipes)

1. Fixed extractor hood

2. Movable extractor hood

3. Extracted work bench

4. Extracted booth

6. Other (please specify)

Annex 3

SOP 3:

Standard operating procedure for blood sampling, including sample storage and transfer to be used in diisocyanates study

WP 8

Task 8.5

Table of Contents

Authors and Acknowledgements 3
1 Introduction 3
2 Material required for blood collection and storage 4
3 Blood Sampling 4
3.1 Blood samples to be collected 4
3.2 Instructions for blood sampling 4
3.3 Sampling procedure and fractionation 5
4 Blood processing 6
5 Sample traceability 6
6 Conservation and storage of the samples 7
7 Transfer of the samples 8
8 Data Reporting 9
9 References 10
Annex 1 - Blood Sampling form 11
Annex 2 - Diagram of blood sampling, fractionating and processing 12
Annex 3 - An illustration of best practices in phlebotomy (WHO, 2010) 13

Authors and Acknowledgements

Lead authors

This document has been developed by Radia Bousoumah and Sophie Ndaw (INRS), France.

Contributions were received from:

Jelle Verdonck (KU Leuven, Belgium), Thomas Göen (IPASUM, Germany), Simo Porras, Tiina Santonen, Sirpa Hyttinen and Riitta Velin (FIOH, Finland).

This document has been created for the HBM4EU project. HBM4EU has received funding from the European Union's Horizon 2020 research and innovation programme under grant agreement No 733032.

1 Introduction

This Standard Operating Procedure (SOP) describes blood sampling for biomonitoring of exposure to hexamethylene diisocyanate (HDI), toluene diisocyanate (TDI), and methylene diphenyl diisocyanate (MDI) under HBM4EU project. Indeed, exposure surveillance is crucial to disease prevention in workplaces where diisocyanates are used.

The approach will be based on the detection of chemical-specific serum immunoglobulin G (IgG) besides haemoglobin (Hb) adducts. In fact, immune mechanisms are believed to be involved in the development of occupational asthma (OA) caused by diisocyanates. Thus, it appears that diisocyanates-specific IgG levels may reflect either exposure or disease.

The main advantages in measuring Hb adducts are (1) to consider cumulative exposures due to the formation of long-lived protein adducts (≤120 days) and (2) the enhanced specificity compared with the HDI/TDI/MDI-related diamines analysis.

In addition to these exposure biomarkers, some effect biomarkers will be investigated. Among them diisocyanate-specific immunoglobulin E (IgE) exhibit high specificity for identifying workers with OA. Markers related to inflammation are also of interest (i.e. IL-6, IL-8, TNF-alpha, IFN-gamma, TIMP-1 and MMP-9)[1].

[1] IL: interleukine, TNF: tumor necrosis factor, IFN: interferon, TIMP: metallopeptidase inhibitor, MMP: matrix metallopeptidase

2 Material required for blood collection and storage

The following materials and equipment will be necessary to undertake the collection of blood samples:

- Phlebotomy syringe with a stainless-steel needle or winged blood sampling set (the vacuum system can be optionally used)
- 70% alcohol swabs for skin disinfection
- Garrottes
- Powder-free disposable gloves
- Tubes with anticoagulant (heparin and EDTA)
- Container for disposal of used needles after venipuncture
- Adhesive bandages or tapes
- Labels
- NaCl solution (0.9%)
- Deionised water
- Bench centrifuge
- Pipettes and tips (1-5 mL)
- Microtubes (2 mL)
- Cryotubes (2 mL)
- Aliquots boxes
- Refrigerator for samples storage at +4ºC
- Freezers for samples storage at -20°C and -80°C
- Cool box
- Dry ice
- Containers appropriate for blood samples shipment (at -20°C and -80°C)
- Blood sampling forms (Annex 1)

3 Blood Sampling

3.1 Blood samples to be collected

Blood sample will be collected from both exposed and non-exposed workers (one sample per participant) at any time during the workweek.

3.2 Instructions for blood sampling

The collection of blood samples requires a clean, quiet and confined space, the availability of sterile material for blood collection and staff trained in phlebotomy knowing the special precautions related to the handling of biological material, according to each country rules.

The trained personnel shall be in charge of the procedure and shall use adequate personal protection equipment (lab coat and gloves).

3.3 Sampling procedure and fractionation

Blood sample is collected by arm vein puncture and manipulated under sterile conditions. The best practices on drawing blood are provided by WHO (2010 - Annex 3). In brief:

- Keep the blood handling area clean and free of dust;
- Use only the supplies provided by the study responsible as detailed in section 2; wear talc-free gloves;
- Prepare the tubes and label them with the code number and other relevant information (date, time of collection);
- Record relevant details in the record form (Annex 1);
- Prepare the volunteer for phlebotomy;
- Place the garrotte in the forearm and disinfect the collection site with 70% alcohol;
- Collect approximately 15 mL of venous blood by phlebotomy, loosen the garrotte and press a cotton ball with 70% alcohol against the puncture site;
- Immediately distribute the blood sample among the two labelled tubes (see Table 1), filling them to the mark to avoid the risk of haemolysis;
- Invert each tube gently 8 times, in order to mix the sample with the anticoagulant;
- Check that the worker is okay and provide a plaster for puncture site as necessary.

Table 1: Fractionation of blood sample according to the analyses to be performed.

		**Tube 1**	**Tube 2**
		**Na Heparin**	**K2 EDTA**
Biomarkers	**Fraction**	**6 mL**	**9 mL**
Diisocyanate specific IgG/IgE and IgE antibodies	Plasma	**X**	
Hb adducts	RBCs		**X**
Inflammatory markers	Plasma	**X**	
	**Volume of blood collected**	**15mL**	

RBCs: red blood cells

A summary scheme for blood distribution among tubes is additionally provided in Annex 2.

4 Blood processing

On arrival in the laboratory, tubes 1 and 2 should be processed to recover the fractions of interest (i.e. plasma and RBCs respectively). Separate plasma and RBC preferably within 8 h of the specimen collection, maximum 24 h, to avoid haemolysis. Specifically (Annex 2):

- Tube 1 is centrifuged for 5 min at 2000 g. The plasma (supernatant) is transferred to microtube and cryotube (approximately 1.5 mL in each tube) and stored under the conditions described in section 6.
- Tube 2 is processed as follows to isolate RBCs [4]:
  - mark the filling level of the blood
  - centrifuge the total blood sample for 5 min at 2000 g (or 10 min at 800 g)
  - remove the plasma layer (or transfer it if need for further investigations)
  - wash RBC fraction three times by filling up to the original filling level with 0.9% sodium chloride (centrifugation and refusal of the aqueous phase)
  - add deionised water until the original filling level
  - store as described in section 6

5 Sample traceability

A standardised convention will be used to assign unique identification codes for all samples collected. The identification code convention is as follows:

Diisocyanate (D) - Country ID (XX) - Company ID (XX) - Participant ID (XXX) - Sample ID (AX/BX/LCX/RCX/BAX/EX/UX/WX)

'D' is to denote that the samples and data relate to the diisocyanate occupational study.

Country ID 'XX' is the country code, using the ISO Alpha-2 country codes for the participating countries (http://www.nationsonline.org/oneworld/country_code_list.htm).

**Country**	**ISO Alpha-2 country codes**
Belgium	BE
Finland	FI
France	FR
The Netherlands	NL
United Kingdom	UK

Company ID 'XX' is a two-digit running number of companies in each country (e.g. 01 for the first company recruited, 02 the second and so forth).

Participant ID 'XXX' is a three-digit running number of participants in each country (e.g. 001 for the first participant recruited, 002 the second and so forth).

Sample ID 'BX' where B denotes the type of sample collected (blood), followed by an one-digit identifier (X) to identify the running number of each type of sample for that worker (e.g. 1 for the first sample, 2 for the second and so forth).

The following scenario is provided to illustrate the application of this convention. A worker is recruited in France. He is working in the first company recruited. He is the first worker recruited in that company and is providing a first blood sample. The sample identification code assigned is therefore D-FR-01-001-B1.

The blood sampling form (Annex 1) - comprising two sections - should be completed for each sample with the following information: company name, worker name and position, date and time of blood collection, identification code (as explained above), number of tubes collected and their destination (according to the type of analysis and Lab that will perform it).

6 Conservation and storage of the samples

Depending on the biomarkers to be analysed, blood samples should be stored until analysis or transfer to a qualified laboratory as follows:

- **Tube 1** (**plasma**) at -20°C (aliquot 1) and at -80°C (aliquot 2)
- **Tube 2** (**RBCs**) at -20°C

Onsite, samples should be kept at +4°C pending transfer to the research team laboratory.

7 Transfer of the samples

Blood samples should be transferred promptly to the research team laboratory (ideally within a few hours after collection) for further processing. For this purpose, tubes should be maintained at +4ºC (by using a cool box).

Regarding shipment to the laboratories that will perform the analyses, destination of tubes and conditions are as follows:

- **Tube 1** (**plasma**) should be shipped to FIOH (aliquot 1) frozen at -20°C and KU Leuven (aliquot 2) frozen at -80°C
- **Tube 2** (**RBCs**) should be sent to IPASUM frozen at -20°C

Table 2 summarises the above-mentioned information for each tube.

Table 2: Transport conditions and destination of tubes.

	**Fraction**	**Biomarker**	**Volume (mL)**	**Transport conditions**	**Prescribed period**	**Destination**
Tube 1	plasma (aliquot 1)	diisocyanate specific IgG/IgE and IgE antibodies	1.5	-20°C	-	FIOH
	plasma (aliquot 2)	inflammatory markers	1.5	-80°C	-	KU Leuven
Tube 2	RBCs	Hb adducts	9	-20°C	-	IPASUM

To ensure transportation at -20°C or -80°C, samples shall be immersed in dry ice using an adequate styrofoam box.

Additionally, the material transfer agreement (MTA) must be signed in advance by both parties (preferably 6 weeks before dispatch). Likewise, a shipping date should be scheduled jointly with the receiver. For this purpose, to avoid being in transit for a long time, especially on weekend, **shipment** should be done on **Monday** and **no later than Wednesday**.

The sample transfer protocol (manifest) must be completed and transmitted with the samples and blood sampling forms [section containing the confidential data (worker identification) must be detached] according to the recommendations referred in D 7.2 “Strategy and SOPs for human sample exchange, including ethical demands”. As soon as dispatched, the recipient is informed by email about the number of samples and estimated date of arrival; the sample data transfer template must be attached.

8 Data Reporting

The following information should be obtained from the laboratory undertaking the analysis which is to be entered into the data template:

- Biomarker concentration (UI/mL [or µg/L], pmol/g Hb)
    - If concentration is below the limit of quantification (LOQ), the result is replaced by <LOQ (for example, <0.23 if 0.23 is the LOQ). Data below LOQ should not be given as an empty cell, zero concentration or free text (i.e. <LOQ, not detected, n.d., LOQ/2)
- Analytical method used
- LOQ of the analytical method (µg/L)
- Method to calculate LOQ

Care must be taken to follow the instructions accompanying the data template. The template must NOT be modified in any way - DO NOT add / remove columns, or alter the drop down lists, or merge cells. In the event that the template is modified or data has been provided which does not follow the instructions, templates will be returned to the data provider for correction.

Below is the list of the biomarkers to be analysed in blood samples.

	**Biomarkers**	**Comments**
Exposure biomarkers	diisocyanates specific IgG	to be completed by FIOH
	Hb adducts	to be performed by IPASUM
Effect biomarkers	diisocyanates specific IgE and IgE antibodies	to be completed by FIOH
	inflammatory markers*	to be performed by KU Leuven

**IL-6, IL-8, TNF-alpha, IFN-gamma, TIMP-1 and MMP-9*

9 References

[1] Dragos M., Jones M., Malo J.-L., Ghezzo H., Gautrin D. Specific antibodies to diisocyanate and work-related respiratory symptoms in apprentice car-painters. *Occup Environ Med* 2009, **66**:227-234.

[2] Flack S.L., Fent K.W., Gaines L.G.T., Thomasen J.M., Whittaker S.G., Ball L.M., and Nylander-French L.A. Hemoglobin adducts in workers exposed to 1,6-hexamethylene diisocyanate. *Biomarkers* 2011, **16**(3): 261-270.

[3] Gui W., Wisnewski A.V., Neamtiu L., Gurzau E., Sparer J.A., Stowe M.H., Liu J., Slade M.D., Rusu O.A., and Redlich C.A. Inception cohort study of workers exposed to toluene diisocyanate at a polyurethane foam factory: initial one-year follow-up. *Am J Ind Med* 2014, **57**:1207-1215.

[4] Sepai O., Henschler D. and Sabbioni G. Albumin adducts, hemoglobin adducts and urinary metabolites in workers exposed to 4,4' -methylenediphenyl diisocyanate. *Carcinogenesis* 1995, **16**(10): 2583-2587.

[5] Sepai O. and Sabbioni G. Albumin adducts and urinary metabolites resulting from occupational exposure to 1,5-naphthalene diisocyanate. *Int J Occup Med Environ Health* 2017, **30**(4):579-591.

[6] Tinnerberg H., Broberg K., Lindh C.H., Jönsson B.A.G. Biomarkers of exposure in Monday morning urine samples as a long-term measure of exposure to aromatic diisocyanates. *Int Arch Occup Environ Health* 2014, **87**:365-372.

[7] Wisnewski A.V., Stowe M.H., Nerlinger A., Opare-Addo P., Decamp D., Kleinsmith C.R. and Redlich C.A. Biomonitoring hexamethylene diisocyanate (HDI) exposure based on serum levels of HDI-specific IgG. *Ann Occup Hyg* 2012, **56**(8), 901-910.

[8] Wisnewski A.V., Liu Q., Liu J., and Redlich C.A. Human innate immune responses to hexamethylene diisocyanate (HDI) and HDI-Albumin Conjugates. *Clin Exp Allergy* 2008, **38**(6): 957-967.

Annex 1 - Blood Sampling form

Confidential Data – do not send with sample

Worker Identification:

Country: ____

Company name and name of department: ____

Worker name: ____

Position: ____

Control? _________ (Yes/No) Exposed? _________ (Yes/No)

--- ◄◄

Send with sample for analysis

Worker Identification:

Blood Sample:

Date: ______________ Time of sampling: _________________________

Blood Code Number: ____

Number of Tubes collected:_____________

Volume of Blood per tube: ______________ml

Destination of Tubes:

1 - ____

Aliquot 1

____

Aliquot 2

2- ____

Notes/Observations:

____

____

____

Annex 2 - Diagram of blood sampling, fractionating and processing

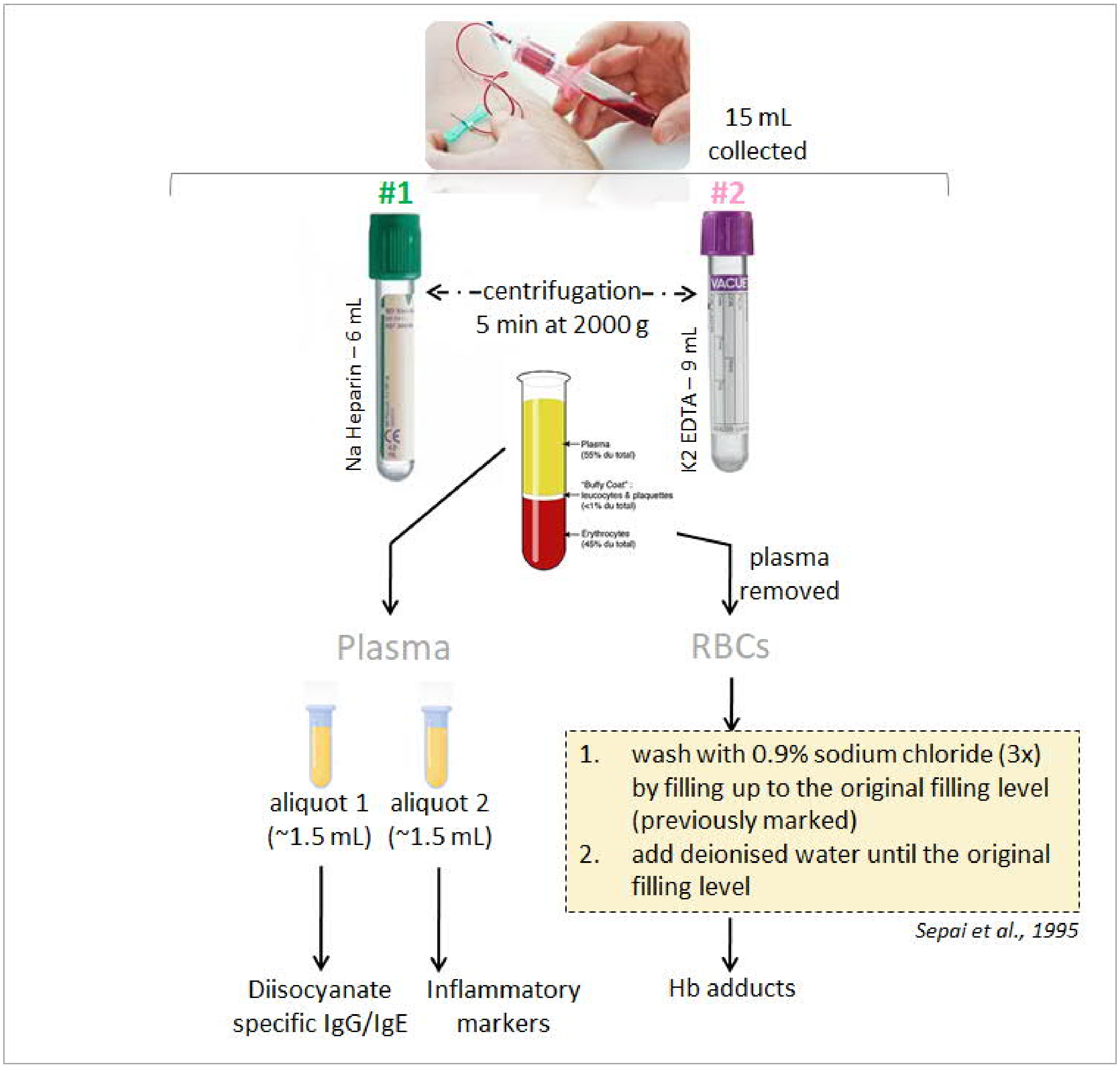

Annex 3 - An illustration of best practices in phlebotomy (WHO, 2010)

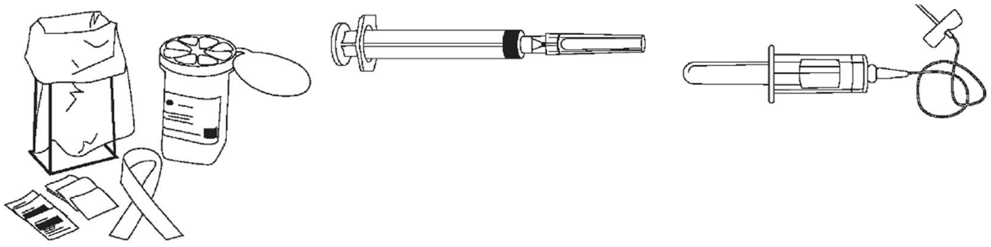

1. Assemble equipment and include needle and syringe or vacuum tube, depending on which is to be used.

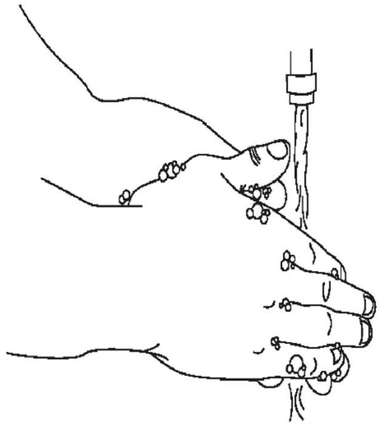

2. Perform hand hygiene (if using soap and water, dry hands with single-use towels).

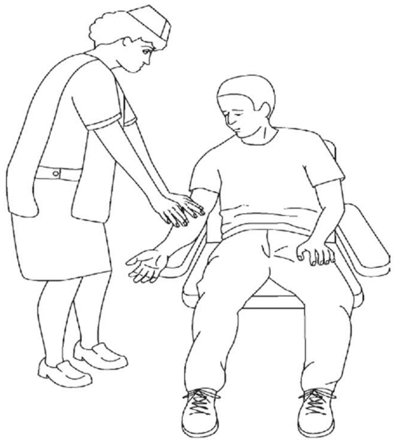

3. Identify and prepare the patient.

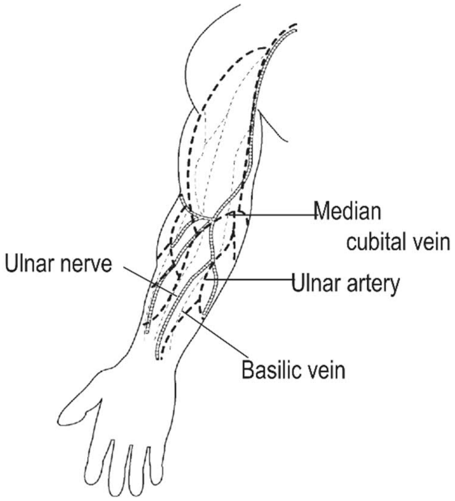

4. Select the site, preferably at the antecubital area (i.e. the bend of the elbow). Warming the arm with a hot pack, or hanging the hand down may make it easier to see the veins. Palpate the area to locate the anatomic landmarks. DO NOT touch the site once alcohol or other antiseptic has been applied.

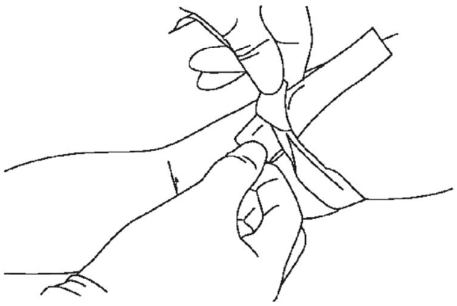

5. Apply a tourniquet, about 4–5 finger widths above the selected venepuncture site.

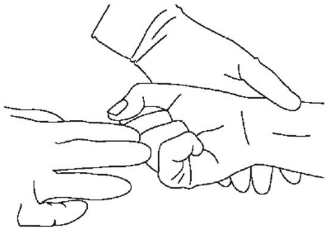

6. Ask the patient to form a fist so that the veins are more prominent.

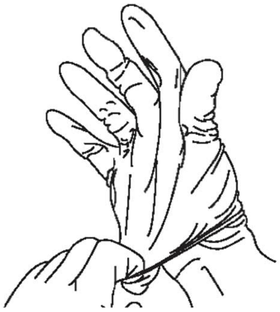

7. Put on well-fitting, non-sterile gloves.

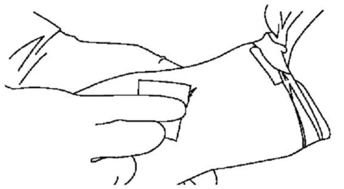

8. Disinfect the site using 70% isopropyl alcohol for 30 seconds and allow to dry completely (30 seconds).

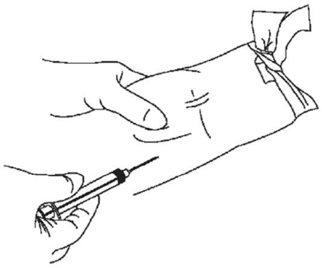

9. Anchor the vein by holding the patient's arm and placing a thumb BELOW the venepuncture site.

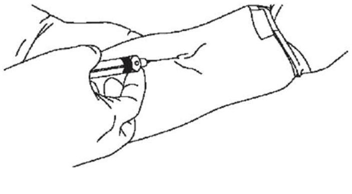

10. Enter the vein swiftly at a 30 degree angle.

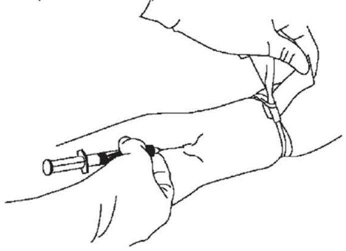

11. Once sufficient blood has been collected, release the tourniquet BEFORE withdrawing the needle.

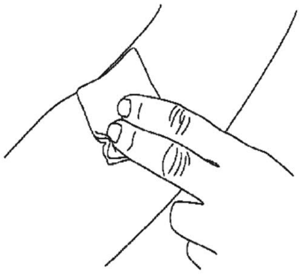

12. Withdraw the needle gently and then give the patient a clean gauze or dry cotton-wool ball to apply to the site with gentle pressure.

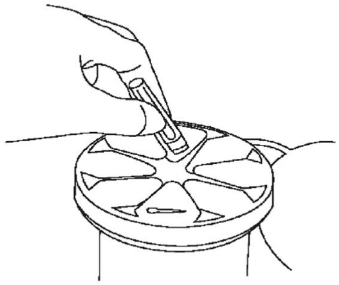

13. Discard the used needle and syringe or blood-sampling device into a puncture-resistant container.

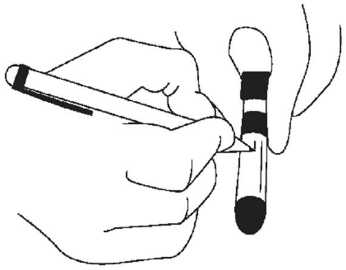

14. Check the label and forms for accuracy.

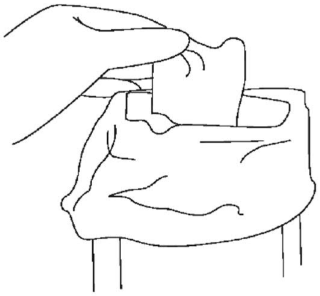

15. Discard sharps and broken glass into the sharps container. Place items that can drip blood or body fluids into the infectious waste.

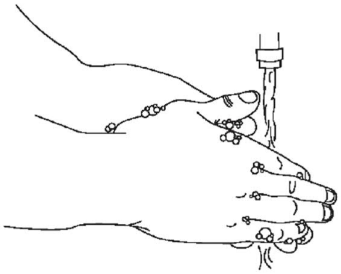

16. Remove gloves and place them in the general waste. Perform hand hygiene. If using soap and water, dry hands with single-use towels.

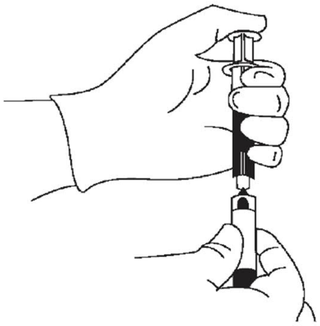

1. If the tube does not have a rubber stopper, press the plunger in slowly to reduce haemolysis (this is safer than removing the needle).

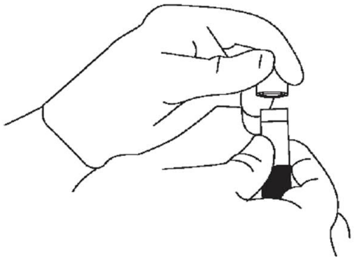

2. Place the stopper in the tube.

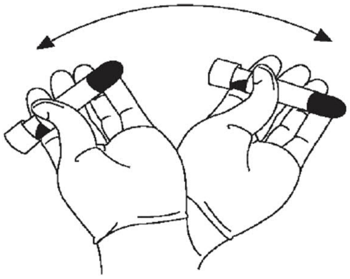

3. Following laboratory instructions, invert the sample gently to mix the additives with the blood before dispatch.

HORIZON2020 Programme
Contract No. 733032 HBM4EU

Annex 4

SOP 4:
Standard operating procedure for urine sampling, including sample storage and transfer to be used in diisocyanates study

WP 8
Task 8.5

Table of Contents

Authors and Acknowledgements 2
1 Introduction 2
2 Material required for urine collection and storage 3
3 Urine sampling 3
3.1 Urine samples to be collected 3
3.2 Information to the workers 3
3.3 Sampling procedure 4
4 Urine processing 4
5 Sample traceability 5
6 Storage of collected urine samples 6
7 Transfer of the samples 6
8 Data reporting 6
9 References 7
Appendix 1: Urine sampling procedure 8
Appendix 2: Sample record sheet 10

Authors and Acknowledgements

Lead authors:

This document has been developed by Radia Bousoumah (INRS, France), Sophie Ndaw (INRS, France), and Kate Jones (HSL, UK).

Contributions were received from:

Riitta Velin and Simo Porras (FIOH, Finland).

This document has been created for the HBM4EU project. HBM4EU has received funding from the European Union's Horizon 2020 research and innovation programme under grant agreement N° 733032.

1 Introduction

This Standard Operating Procedure (SOP) describes urine sampling for biomonitoring of exposure to hexamethylene diisocyanate (HDI), toluene diisocyanate (TDI), and methylene diphenyl diisocyanate (MDI), the most commonly used diisocyanates in Europe.

Biomonitoring holds several advantages in exposure assessment (1) sampling is convenient, (2) integrates all routes of exposure and (3) biomarkers with long half-times, such as protein adducts, may be used to monitor long-term exposure. Thus TDI/MDI-lysine adducts will be analysed in this study in order to investigate the diisocyanate specific metabolites from the urine alongside the analysis of the commonly used biomarkers characterized by a short half-life and lack of specificity [i.e. HDI/TDI/MDI-related metabolites released as hexamethylene diamine (HDA), 2,4- and 2,6-toluene diamine (2,4-TDA and 2,6-TDA respectively) and methylenediphenyl diamine (MDA)].

These specific biomarkers may better correlate with systemic adverse health effects, such as sensitization enabling a better risk assessment to diisocyanates.

2 Material required for urine collection and storage

To undertake the collection of urine samples, researchers should ensure that they have sufficient quantities of the following:

- Wide-mouth polypropylene bottles (100 mL or more) for urine collection
- Urine aliquot containers (30 mL polypropylene vessel with a screw cap)
- Urine aliquot containers (30 mL polypropylene vessel with a screw cap, containing 0.5 g citric acid)
- Citric acid (as a preservative)
- Labels
- Biological and hermetic bags
- Dark coloured bags
- Hand washing and sampling procedures (Appendix 1)
- Nitrile or similar disposable gloves
- Graduated cylinder (capacity 25 mL)
- Freezers
- Dry ice
- Containers for urine samples shipment
- Trash cans
- Pens
- Sample record sheets (Appendix 2)

3 Urine sampling

3.1 Urine samples to be collected

Urine samples are collected three times (1) before the work shift (pre-shift void)[1], (2) at the end of the work shift (post-shift void), and (3) the next day in the morning (first morning void).

For the control group (non-occupationally exposed workers) only one sample is collected in the post-shift.

3.2 Information to the workers

To deliver spot urine samples, workers are asked to remove their work clothes (overalls) and to wash their hands thoroughly with soap and water before voiding to avoid dust failing into the sampling container (e.g. from cloth and skin). To this end, printed instructions on the procedure and hygiene of urine collection are delivered to each participant (Appendix 1).

Following collection, date and time are registered by the participants on the container’s label and the samples are returned to the research team.

[1] On Tuesday, Wednesday or Thursday morning.

3.3 Sampling procedure

Three urine samples are collected by the workers starting from pre-shift on the measurement day until the morning of the next day. In brief:

- Name labelled urine containers are delivered to each participant, along with biological hermetic bags to place the container inside.
- Before the sampling, the participant completes the label with the required information (date, hour). Once done, he removes his work clothes (overalls) and washes his hands with soap and water according to the provided instructions (Appendix 1).
- The participant opens the urine collection container, collects his urine, screws the cap firmly and places the container in the biological and hermetic bag provided to avoid any leak.
- The participant returns the urine collection container to the research team. For the transport from home to work (i.e. first urine of the next day), dark coloured bags are left at disposal by the research team.
- The researcher checks that the required label details are recorded and that these are correct and legible.

4 Urine processing

Whenever possible, urine samples should be conditioned by the HBM4EU research team onsite to prevent degradation of the TDA (citric acid used as a preservative). The processing must be carried out in an area free from contamination e.g. office, medical service, etc. The wear of disposable gloves is mandatory. The researcher should also ensure that health and safety precautions are applied.
Aliquots are prepared as follows:

- 1 aliquot of 20 mL of urine in a 30 mL container containing 0.5 g citric acid[2], for diamines (e.g. MDA, TDA, HDA) and creatinine analyses;
- 1 aliquot of 20 mL of urine in plain 30 mL container, for lysine-adducts analyses (and TDA comparison)[3];
- 1 aliquot of 20 mL of urine in plain 30 mL container for preservation of samples in each respective institute.

Urine collection containers are thrown in the trash can. Ideally, the research team collect all the used trash cans to dispose of them according to their standard practice.

[2] Labeled "with citric acid" in order to distinguish those containers containing citric acid from those without (i.e. next aliquots).
[3] Only the Labs who are interested to investigate the need for citric acid as a preservative for TDA since the stability is generally tested on spiked samples. It is an opportunity to compare acidified urine *versus* plain urine side by side. In this case, the aliquot of 20 mL should be split into 2 aliquots of 10 mL (provide 10 mL polypropylene vessel). One aliquot is sent to HSL for lysine analysis and the other is retained by the researchers for TDA analysis where appropriate.

5 Sample traceability

The aliquot containers are properly labelled with the sample code. A standardised convention will be used to assign unique identification codes for all samples collected. The identification code convention is as follows:

Diisocyanate (D) - Country ID (XX) - Company ID (XX) - Participant ID (XXX) - Sample ID (AX/BX/LCX/RCX/BAX/EX/UX/WX)

'D' is to denote that the samples and data relate to the diisocyanate occupational study.

Country ID 'XX' is the country code, using the ISO Alpha-2 country codes for the participating countries (http://www.nationsonline.org/oneworld/country_code_list.htm).

Country	ISO Alpha-2 country codes
Belgium	BE
Finland	FI
France	FR
The Netherlands	NL
United Kingdom	UK

Company ID 'XX' is a two-digit running number of companies in each country (e.g. 01 for the first company recruited, 02 the second and so forth).

Participant ID 'XXX' is a three-digit running number of participants in each country (e.g. 001 for the first participant recruited, 002 the second and so forth).

Sample ID 'UX' where U denotes the type of sample collected (urine), followed by a one-digit identifier (X) to identify the running number of each type of sample for that worker (e.g. 1 for the first sample, 2 for the second and so forth).

The following scenario is provided to illustrate the application of this convention. A worker is recruited in France. He is working in the first company recruited. He is the first worker recruited in that company and is providing a first urine sample. The sample identification code assigned is therefore D-FR-01-001-U1.

The sample record sheet (Appendix 2) should be completed for each urine sample in order to confirm collection of the required aliquots. Following items are recorded: identification code (as explained above), name of the participant, sampling date, time of collection, aliquots available and information related to the company (name, ID) and researcher (name, institute).

6 Storage of collected urine samples

Aliquoted samples should be stored under frozen (-20°C) conditions until analysis or transfer to a qualified laboratory (i.e. passed Anilines ICI EQUAS rounds) to perform chemical analysis in HBM4EU.

7 Transfer of the samples

Urine aliquoted samples will be sent to the research team laboratory or laboratories that will perform the analyses by maintaining the cold chain, by using freezer or dry ice.

Regarding samples that should be transferred to another laboratory for analyses, the material transfer agreement (MTA) must be signed in advance by both parties (preferably 6 weeks before dispatch). Likewise, a shipping date should be scheduled jointly with the receiver [i.e. qualified as HBM4EU laboratory for diamines (as a result of the interlaboratory comparison rounds for anilines in urine) and HSL for MDI/TDI-lysine].

The sample transfer protocol (manifest) must be completed and transmitted with the samples according to the recommendations referred in D 7.2 “Strategy and SOPs for human sample exchange, including ethical demands”. As soon as dispatched, the recipient is informed by email about the number of samples and estimated date of arrival; the sample data transfer template must be attached.

8 Data reporting

The laboratory performing the analyses reports:

- Urinary concentration of samples (µg/L)
    - If concentration is below the limit of quantification (LOQ), the result is replaced by **<**LOQ (for example, **<0.23** if 0.23 is the LOQ). Data below LOQ should not be given as an empty cell, zero concentration or free text (i.e. <LOQ, not detected, n.d., LOQ/2) - please ensure that the **<** is used to identify the result as <LOQ.
- Analytical method used
- LOQ of the analytical method (µg/L)
- Method to calculate the LOQ (the laboratory should test that the reported LOQ concentration can be analysed accurately (RSD <20 %))
- Creatinine concentration of each urine sample (g/L)
- Method to determine creatinine and LOQ of the method (g/L)

Below is the list of the biomarkers to be analysed in urine samples.

Biomarkers	Comments
Diamines (HDA/TDA/MDA)	
Creatinine	
MDI/TDI-lysine	To be completed by HSL

9 References

1. Budnik L.T., Nowak D., Merget R., Lemiere C. and Baur X. Elimination kinetics of diisocyanates after specific inhalative challenges in humans: mass spectrometry analysis, as a basis for biomonitoring strategies. *J Occup Med Toxicol* 2011, **6**:9.

2. Cocker J., Jones K., Leng G., Gries W., Budnik L.T., Müller J., Göen T., Hartwig A., MAK Commission. Hexamethylene diisocyanate, 2,4-toluene diisocyanate, 2,6-toluene diisocyanate, isophorone diisocyanate and 4,4′-methylene diphenyl diisocyanate – Determination of hexamethylenediamine, 2,4-toluenediamine, 2,6-toluenediamine, isophoronediamine and 4,4′-methylenedianiline in urine using gas chromatography-mass spectrometry. *The MAK Collection for Occupational Health and Safety* 2017, **2**:3.

3. Hu J., Cantrell P. and Nand A. Comprehensive Biological Monitoring to Assess Isocyanates and Solvents Exposure in the NSW Australia Motor Vehicle Repair Industry. *Ann Work Expos Heal* 2017, **61**(8) :1015-1023.

4. Littorin M., Axmon A., Broberg K., Sennbro C.J., Tinnerberg H. Eye and airway symptoms in low occupational exposure to toluene diisocyanate. *Scand J Work Environ Health* 2007, **33**(4):280-285.

5. Mirmohammadi S., Ibrahim M.H. and Saraji G.N. Evaluation of hexamethylene diisocyanate as an indoor air pollutant and biological assessment of hexamethylene diamine in the polyurethane Factories. *Advanced Air Pollution* 2011, Dr. Farhad Nejadkoorki (Ed.), ISBN: 978-953-307-511-2, InTech, Available from: http://www.intechopen.com/books/advanced-air-pollution/evaluation-ofhexamethylene-diisocyanate-as-an-indoor-air-pollutant-and-biological-assessment-of-hex

6. Pronk A., Yu F., Vlaanderen J., Tielemans E., Preller L., Bobeldijk I., Deddens J.A., Latza U., Baur X., Heederik D. Dermal, inhalation, and internal exposure to 1,6-HDI and its oligomers in car body repair shop workers and industrial spray painters. *Occup Environ Med* 2006, **63**:624-631.

7. Robert A., Ducos P., Francin J.-M., Marsan P. Exposition au MDI dans 19 entreprises françaises – La MDA urinaire comme biomarqueur de l'exposition. INRS - *Hygiène et Sécurité au Travail* 2007.

8. Sennbro C.J., Lindh C.H., Tinnerberg H., Welinder H., Littorin M., Jönsson B.A.G. Biological monitoring of exposure to toluene diisocyanate. *Scand J Work Environ Health* 2004, **30**(5):371-378.

9. Williams N.R., K Jones K., Cocker J. Biological monitoring to assess exposure from use of isocyanates in motor vehicle repair. *Occup Environ Med* 1999, **56**:598-601.

Appendix 1: Urine sampling procedure

Urine sample and hand washing procedure

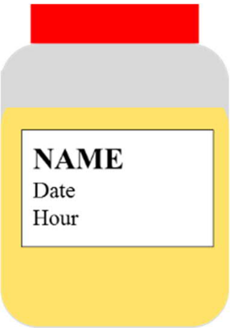

1. Take a urine collection container and biological bag from the research team

2. Complete the label with your name, date and time of collection

3. Carefully wash your hands following the instructions below

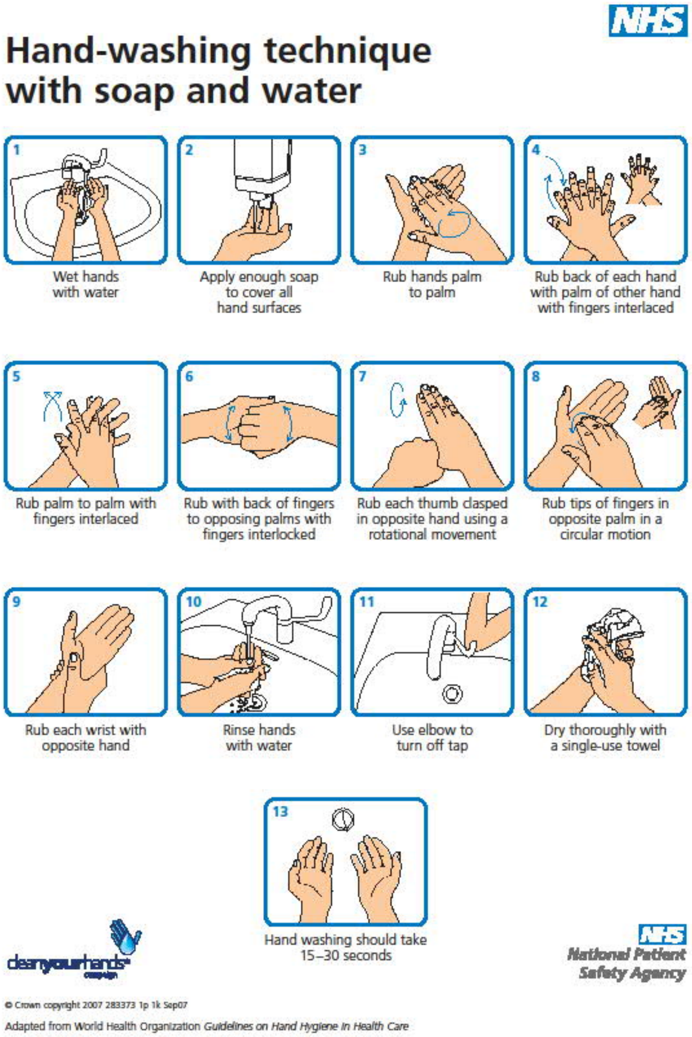

4. Open the container and collect your urine

5. Screw on the cap and place container in the biological bag

6. Immediately return the sample to the research team

Appendix 2: Sample record sheet

**Company Name:**	
**Company ID:**	

**Researcher(s):**	
**Research Organisation:**	

**Sample ID**	**Name**	**Date**	**Hour**	**Aliquots collected for further analysis and storage**			**Remarks**
				1 x 20 mL diamines and creatinine	1 x 20 mL lysine adducts[4]	1 x 20 mL preservation	

[4] If the aliquot is splited into 2 aliquots of 10 mL in order to investigate the need for citric acid as a preservative for TDA, please specify this in the remarks column.

HORIZON2020 Programme
Contract No. 733032 HBM4EU

Annex 5

SOP 5:

Standard operating procedure for assessing dermal exposure to diisocyanates

WP 8

Task 8.5

Table of Contents

Authors and Acknowledgements 2

1 Introduction 2

2 When should wipe samples be collected? 3

3 Materials required for the collection of wipe samples 3

4 Participant instructions 4

5 Researcher precautions 4

6 Wipe sampling procedures 4

7 Frequency of sample collection 5

8 Sample traceability and contextual information 6

9 Storage of photographs 6

10 Quality control 7

11 Reporting 7

References 7

Appendix 1: Sample record sheet 8

Appendix 2: Semi-quantitative assessment of diisocyanates exposure 10

Authors and Acknowledgements

Lead authors:

This document has been developed by Dr Karen Galea (IOM, UK) and Kate Jones (HSE, UK).

Contributions were received from:

Glen McConnachie, Matthew Coldwell (HSE), Marika Loikala (FIOH, Finland), Lode Godderis, Jelle Verdonck, Katrien Poels (KU Leuven, Belgium), Radia Bousoumah and Sophie Ndaw (INRS, France).

This document has been created for the HBM4EU project. HBM4EU has received funding from the European Union's Horizon 2020 research and innovation programme under grant agreement No 733032.

1 Introduction

This Standard Operating Procedure (SOP) is focussed on the assessment of dermal exposure to diisocyanates. This will be undertaken using Colorimetric SWYPE™ indicators[1] which are a simple and sensitive technique to detect contaminants on the skin (and also work surfaces). In brief, the hands will be wiped with a SWYPE™ which is then treated with a developing solution. A rapid, positive colour change occurs if the contaminant is present, with the resulting colour change giving a semi-quantitative assessment of exposure which can be recorded.

This SOP has been developed based on materials published by SKC (SKC, 2015), OSHA (OSHA, 1997) as well as key publications from the peer-reviewed literature (e.g. Ceballos et al, 2009). This SOP was also developed with due consideration of the contents of the "SOP7: Standard operating procedure for obtaining dermal samples" (lead authors Dr. K Galea and Prof. JW Cherrie, both IOM) developed for the HBM4EU occupational biomonitoring study on hexavalent chromium and other harmful chemicals (Porras et al., 2019).

[1] https://www.skcltd.com/products2/surface-skin-sampling/colorimetric-swypes.html

2 When should wipe samples be collected?

It is proposed that wipe sampling will only take place during tasks when dermal exposure is likely. Table 1 indicates where wipe sampling may be useful, depending on the activities being undertaken and considered within this programme of work.

Pre- and post- task based sampling is intended and it is important for the field researcher to record details of the task being assessed in the sample record sheet (Appendix 1).

Table 1: Activities being considered under programme of work and indication of whether wipe sampling will be necessary

Activity	Wipe sampling necessary?
Use of diisocyanate based glues, adhesives or sealants	Yes
Coating large surfaces (e.g. floors) with polyurethane coatings	Yes
Spray application of urethane foam in construction or boat manufacturing sector	Yes
Spray coating of large vehicles with di-isocyanate containing paints and primers	Yes
Welding, grinding and flame cutting of polyurethane painted materials or e.g. polyurethane-insulated district heating pipes	No

3 Materials required for the collection of wipe samples

It is recommended that researchers liaise with the site prior to the sampling campaign to establish whether aromatic or aliphatic diisocyanates are being used. To undertake the collection of wipe samples, researchers should ensure that they have sufficient quantities of the following:

- Skin SWYPEs, Aromatic Isocyanates, pk 20 (Part No. 769-1032)
- Skin SWYPEs, Aliphatic Isocyanates, pk 20 (Part No. 769-1033)
- Cleaner/Developer, Aromatic and Aliphatic Isocyanates, spray bottle (Part No. 769-1062).
- Supply of disposal nitrile gloves
- Sample record sheets (Appendix 1)
- Laminated colour copy SWYPE, semi-quantitative assessment (Appendix 2). Laminated copies will be made available from the study co-ordinator and issued to each field team to ensure consistency in printing.
- Pens
- Digital Camera / mobile phone with camera
- Receptacle for disposing of collected samples

4 Participant instructions

Participants should be fully informed of the sampling procedure. They will be advised of the following points before commencing their work shift:

- Participants will be asked to thoroughly wash and dry their hands with soap and water before commencing their work shift. Hands will be dried using fresh disposable paper towels (which can be provided as necessary). Reusable fabric towels must not be used.
- If protective gloves are normally worn by the worker, request that fresh, unused gloves are worn if possible. If reusable gloves are being worn this should be recorded (Appendix 1).
- Participants will be informed that wipe samples will be collected prior to their task involving diisocyanates (e.g. spray job) and then again just after this is completed.
- Participants will be requested not wash their hands after the task involving diisocyanates is completed and before wipe samples are collected. If for any reason they do wash their hands between any two wipe sampling periods, they should inform the researcher that this is the case. The time between washing and wiping should be noted.

5 Researcher precautions

When collecting each wipe sample from the participants, researchers must always wear a new pair of disposable nitrile gloves.

Care should also be taken to avoid cross contamination of samples. It is recomended that where possible the wipes are collected in an area considered to be free of potential contamination e.g. office space, medical room etc. Wipes should not be collected in the physical work area where diisocyanates are present due to the risk of cross contamination. It is also important that researchers ensure that the sampling location itself does not become contaminated with diisocyanates.

Researchers collecting the wipe samples should try to ensure that a consistent amount of pressure is applied when wiping the participants hand.

Researchers should ensure that a site specific risk assessment of their work practices is undertaken prior to commencing the measurement campaign and that all necessary health and safety precautions are adopted and followed.

6 Wipe sampling procedures

Before sampling it is important to establish what type of diisocyanates are being used in the activity so that the correct Skin SWYPE™ pad (aromatic or aliphatic) is selected for use in the sampling.

The following procedures should be used to collect the wipe samples.

- Wear a new pair of clean disposable gloves for each sample.
- Ask the participant what their dominant hand is.
- Ask the participant if they have washed their hands before the samples being collected. If yes, note the time elapsed.

- Wipe the skin of the palmer region of the dominat hand (Figure 1) with the cloth portion of the selected Skin SWYPE™ pad (aromatic or aliphatic). The region must be wiped three times in a circular manner.

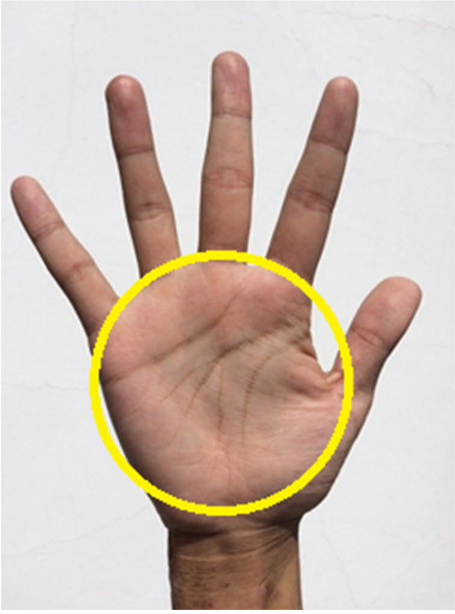

Figure 1: Area of palmar region of the hand to be wiped (highlighted by red circle)

- Pour ¼" (~6.4 mm) of developing solution into the small cup (provided with the kit).
- Put the Skin SWYPE™ in the cup, cloth end down and colour detection strip up.
- The developing solution will wick up to the colour detection strip and a colour change will occur if contamination is present.
- After exactly 4 minutes, record the colour change in the sample record sheet (Appendix 1) comparing this with the laminated copy of Figure 2.
- Take a photograph of the Skin SWYPE™, positioned next to although not touching the laminated copy of Appendix 2, noting down the sample ID and time photograph was taken in the sample record sheet (Appendix 1).

7 Frequency of sample collection

The number of wipe samples collected will be dependent on the number of relevant tasks undertaken during the shift and acceptability of the process by the worker. Care should also be taken so not to overly intrude on the workers normal shift work.

8 Sample traceability and contextual information

Although no samples are being sent to a laboratory for analysis, images of the Skin SWYPE™ are being collected as this will allow different reseachers to compare their subjective assessments and ensure standardization of the assessments. A standardised convention should therefore be used to assign unique identification codes for these images. The identification code convention is as follows:

Diisocyanate (D) - Country ID (XX) - Company ID (XX) - Participant ID (XXX) - Sample ID (AX/BX/LCX/RCX/BAX/EX/UX/WX)

'D' is to denote that the samples and data relate to the diisocyanate occupational study.

Country ID 'XX' is the country code, using the ISO Alpha-2 country codes for the participating countries (http://www.nationsonline.org/oneworld/country_code_list.htm).

**Country**	**ISO Alpha-2 country codes**
Belgium	BE
Finland	FI
France	FR
The Netherlands	NL
United Kingdom	UK

Company ID 'XX' is a two-digit running number of companies in each country (e.g. 01 for the first company recruited, 02 the second and so forth).

Participant ID 'XXX' is a three-digit running number of participants in each country (e.g. 001 for the first participant recruited, 002 the second and so forth).

Sample ID 'WX' where W denotes the type of sample collected (wipe), followed by an one-digit identifier (X) to identify the running number of each type of sample for that worker (e.g. 1 for the first sample, 2 for the second and so forth).

The following scenario is provided to illustrate the application of this convention. A worker is recruited in UK. He is working in the first company recruited. He is the first worker recruited in that company and is providing a first wipe sample which has been photographed. The photograph code assigned is therefore D-UK-01-001-W1

9 Storage of photographs

The collected images should be downloaded from the camera / mobile phone upon return to the laboratory, assigned the unique sample code (Section 8) and stored securely as per other electronic files pertaining to the project.

10 Quality control

A round robin exercise will be held to assess the level of agreement with coding the Skin SWYPE™ colours between the field researchers. It is intended that this will be undertaken on at least two occasions, the first being shortly after the start of the sampling campaign and the second being half way through.

In preparation for each round robin, the main researcher from each participating country will be asked to provide 5 images (selected at random) collected from study participants (therefore 25 images in total). These images will then be issued to each of the field researchers who will be asked to code the images using the scheme in Appendix 2. The returned coding will be assessed for level of agreement between the researchers, with a 90% agreement being judged as acceptable.

The round robin scheme will be managed by Ms Kate Jones (HSE).

11 Reporting

It is important that information entered into the sample record sheet (Appendix 1) is recorded as per the instructions given in this SOP as this information will need to be entered into the data template for the overall di-isocyanate study. With respect to the wipe result a simple numeric value (0-3) is all that is required.

Care must be taken to follow the instructions accompanying the data template. The structure, drop down lists etc of the data template must NOT be modified in any way. In the event that the template is modified or data has been provided which does not follow the instructions, templates will be returned to the data provider for correction.

References

Ceballos DM, Yost MG, Whittaker SG, Camp J, Dills R. (2009) Objective Color Scale for the SWYPE Surface Sampling Technique Using Computerized Image Analysis Tools'. Journal of Occupational and Environmental Hygiene; 6(10): 604-611. Available from URL: https://doi.org/10.1080/15459620903117710

Porras S, Ladeira C, Ribeiro E, Viegas S, Uuksulainen S, Santonen T, Galea K, Cherrie J, Louro H, Ventura C, Silva MJ, Leese E, Jones K, Hanser O, Ndaw S, Robert A, Duca R-C, Poels K, Godderis L, Kiilunen M, Norppa H, Veijalainen H, Parshintsev E, Tuomi T, Ruggieri F, Alimonti A, Koch H, Bousoumah R, Antoine G, Jacoby N, Musgrove D. (2019) HBM4EU occupational biomonitoring study on hexavalent chromium and other harmful chemicals. Standard Operating Procedures (SOPs). WP8 - Targeted field work surveys and alignment at EU level. Available from URL: https://www.hbm4eu.eu/online-library/ (last accessed 27th January 2021).

OSHA (1997) Aromatic Isocyanate Surface Contamination Sampling and Evaluation Techniques. Available from URL: https://www.osha.gov/SLTC/isocyanates/mrl_inte.html (last accessed 27th January 2021).

SKC (2015) Colorimetric SWYPEs. Available from URL: https://www.skcltd.com/products2/surface-skin-sampling/colorimetric-swypes.html#skin-swypes (last accessed 27th January 2021).

Appendix 1: Sample record sheet

**Company Name:**	
**Company ID:**	
**Worker ID:**	
**Name (family name, initial):**	
**Job title:**	
**Dominant hand - Right or Left:**	
**Gloves worn – fresh or reused?:**	
**Type of gloves worn:**	

**Researcher(s):**	
**Organisation:**	
**Date sampling:**	

**Collection time (00:00)**	**Sample ID**	**Pre- or post-task?**	**Time photograph taken (00:00)**	**Gloves worn prior to collection (Y/N)**	**Hands washed prior to collection (Y/N)**	**If yes, time (mins) since hands were last washed**	**Colour change code**

Task details (free text description of task):

**Did the task include the following?**	**Duration of task (mins)**	**Diisocyanates used and quantities (litres)**	**Subjective assessment of dermal exposure occurred (Yes/ No)**

	**Yes / No**
Filling, mixing or loading	
Wiping	
Dispersion hand-held tools	
Spraying	
Immersion	
Mechanical treatment	

	Y/N
Dermal exposure unlikely to have occurred	
Direct contact with the substance	
Contact to airborne material	
Contact with contaminated surfaces	

Appendix 2: Semi-quantitative assessment of diisocyanates exposure

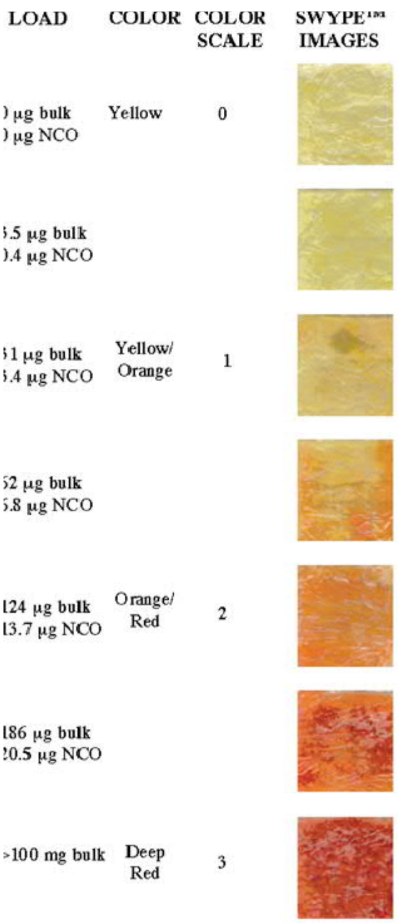

LOAD	COLOR	COLOR SCALE	SWYPE™ IMAGES
[illegible] µg bulk<br>[illegible] µg NCO	Yellow	0	
[illegible].5 µg bulk<br>[illegible].4 µg NCO			
[illegible]1 µg bulk<br>[illegible].4 µg NCO	Yellow/ Orange	1	
[illegible]2 µg bulk<br>[illegible].8 µg NCO			
[illegible]24 µg bulk<br>[illegible]3.7 µg NCO	Orange/ Red	2	
[illegible]86 µg bulk<br>[illegible]0.5 µg NCO			
[illegible]100 mg bulk	Deep Red	3	

HMB4EU code to be applied
0
1
2
3

HORIZON2020 Programme
Contract No. 733032 HBM4EU

Annex 6

SOP 6:

Standard operating procedure for air sampling of diisocyanates

WP 8

Task 8.5

Table of Contents

Authors and Acknowledgements 2
1 Scope 2
2 When should air samples be collected? 4
3 Materials required for the collection of air samples 5
4 Recommendations and precautions for air sampling 5
5 Air sampling procedure 6
6 Sample traceability and contextual information 7
7 Storage of collected air samples 8
8 Quality control 8
9 Transportation of air samples to laboratory 9
10 Data reporting 9
References 10
Appendix 1: Sample record sheet 11

Authors and Acknowledgements

Lead authors:

This document has been developed by Dr Karen Galea (IOM, UK) and Kate Jones (HSE, UK).

Contributions were received from:

Glen McConnachie, Matt Coldwell (HSE), Marika Loikala (FIOH, Finland), Lode Godderis, Jelle Verdonck, Katrien Poels (KU Leuven, Belgium), Radia Bousoumah and Sophie Ndaw (INRS, France).

This SOP was also developed with due consideration of the contents of the "SOP6: Standard operating procedure for air sampling of inhalable and respirable dust fraction and (hexavalent) chromium (SOP 6)" (lead authors Dr Radu-Corneliu Duca, Dr Katrien Poels and Prof. Lode Godderis (KU Leuven, Belgium) developed for the HBM4EU occupational biomonitoring study on hexavalent chromium and other harmful chemicals (Porras et al, 2019).

This document has been created for the HBM4EU project. HBM4EU has received funding from the European Union's Horizon 2020 research and innovation programme under grant agreement No 733032.

1 Scope

This Standard Operating Procedure (SOP) is for the collection of workers personal exposure to airborne diisocyanates. The sampling methodology will utilise Supelco ASSET™EZ4 Dry Samplers for Isocyanates (Asset EZ4-NCO) which allow for the collection of a wide range of isocyanate monomers and oligomers (Brown et al). This is a relatively new active sampling device which is a dry sampler based on derivatization of isocyanate groups with di-n-butylamine (DBA). The sampler consists of a denuder (syringe barrel) and a filter cassette, both impregnated with DBA. Vapor phase isocyanates are collected in the denuder and the isocyanate particles are collected on the filter.

Other air sampling methods for isocyanates are available. Sampling using a reagent impregnated glass fibre filter in combination with liquid reagent filled glass impinger is common, particularly where aerosols as well as vapours are present. Where only vapours are present a reagent impregnated glass fibre filter may be used without an impinger. Impingers are not recommended for personal sampling as they may leak harmful solvents or the glass may break. While the filter only techniques can use a high sampling rate of 2 litres per minute compared to the standard 200 ml per minute of the Asset EZ4-NCO sampler, they may require stabilising with additional reagent immediately after sampling.

The Asset EZ4-NCO has been selected for use in the HMB4EU diisocyanates sampling campaign as easier and more practicable to use than some other available methods and it allows standardization of air sampling across the project. It is important to highlight however that the method does have some limitations in that it is not a total-NCO method and there may be issues with sampling for aliphatic diisocyanates, for aerosols and for short activities due to the reduced sample volume. To compensate for the latter issue, we are recommending that a flowrate of 850 mL/min is used for short activities (15 minutes) otherwise a flowrate of 250 mL/min should be used.

Unfortunately the samples can only be analysed by (Liquid Chromatography Mass Spectrometry), LC-MS-MS (Liquid Chromatography tandem Mass Spectrometry) or CLND (ChemiLuminescent Nitrogen Detection). The reagent has no UV chromophore and so it is only possible to detect the isocyanates the instrument has been specifically set up for. The study will focus on analyzing the collected samples for MDI, TDI and HDI but importantly, not just for monomers. It is therefore crucial to know what isocyanates are used in the processes being monitored and so it is important that good contextual information is gathered during the sampling campaign with respect to this which is then passed onto the analytical laboratory (Appendix 1). Ideally product material safety data sheets will be collected (or accessed afterwards from good product identification on site).

Sigma-Aldrich has a freely available PowerPoint presentation discussing the Asset EZ4-NCO sampler, sampling and analytical requirements (Michel and Schultz, 2012). This is available at

https://www.sigmaaldrich.com/content/dam/sigma-aldrich/docs/Supelco/General_Information/1/06_Isocyanate%20Sampling.pdf

It is strongly recommended that this is accessed and printed to accompany this SOP for completeness[1].

For those undertaking the laboratory analysis, Merck KGaA (2020) provides details of the certified reference materials developed for use with the Asset EZ4-NCO sampler. The “Matrix Solution” required for the calibration standards is prepared in a 100 mL flask by adding together ~60 mL methanol, 25 mL dibutylamine and then slowly adding 8.32 mL of acetic acid while stirring the solution. Finally add methanol to the mark on the flask.

A full guide to the extraction and analysis procedure (Extraction and Analysis of ASSET™EZ4-NCO Dry Sampler Isocyanate Monomers Guide, Rev 2.0) is available on request from Kristen Schultz (Product Manager) kristen.schultz@sial.com. A presentation on the extraction procedure is available at

https://www.sigmaaldrich.com/content/dam/sigma-aldrich/docs/Supelco/Instructions/1/ASSET_EZ4-NCO_Extraction.pdf

[1] Pdf presentation not included within this SOP due to copyright restrictions.

2 When should air samples be collected?

It is proposed that air sampling will only take place during activities where aerosols are likely. Table 1 indicates where air sampling may be useful, depending on the activities being undertaken and considered within this programme of work.

Table 1: Activities being considered under programme of work and indication of whether air sampling will be necessary, subject to a local assessment depending on conditions and use of RPE etc.

Activity	Air sampling necessary?
Use of diisocyanate based glues, adhesives or sealants	No, although consider where heat is significant
Coating large surfaces (e.g. floors) with polyurethane coatings	Case by case basis
Spray application of urethane foam in construction or boat manufacturing sector	Yes
Spray coating of large vehicles with di-isocyanate containing paints and primers	Case by case basis.*
Welding, grinding and flame cutting of polyurethane painted materials or e.g. polyurethane-insulated district heating pipes	Yes

* If using temporary enclosures then within enclosure and outside if any bystanders without PPE within 10m

It is important for the field researcher to record details of the activity being assessed in the sample record sheet (Appendix 1).

Given the limited number of air samples available per country within the HMB4EU budget and to minimise intrusion on the participants' work, it is recommended that no more than one activity based air sample is collected from each worker (where air sampling is considered necessary). To allow for an estimate of the workers 8-hr time weighted average (TWA) to be determined (at the data analysis stage) details of the number of instances that the monitored di-isocyanate related activity is undertaken during the work shift should be recorded in the record sheet. In the event of the activity lasting a significant period of the work shift, it is recommended that the duration of the measurement period does not exceed 4 hours.

3 Materials required for the collection of air samples

Below is a list with the different materials needed for performing air measurements.

- AssetEZ4 dry sampler – available from Sigma Aldrich in packs of 10 or 50 samplers (www.sigmaaldrich.com)
- Calibration fitting (supplied with each pack of samplers)
- Adaptors to secure sampler to tubing (some are supplied with each pack of samplers)
- Low flow air pumps, capable of operating 250 ml/min for 4 hours or 850 ml/min for short-duration activities.
- Flow rate calibrator, e.g. Defender 510 (BIOS DryCal), calibrated against a primary standard, capable of measuring the required flow rates
- Protective pump pouches and belts / harnesses to allow sampling equipment to be attached to wearer
- Supply of clips to attach samplers to participants
- Sufficient lengths of Tygon flexible tubing of suitable diameter for making a leak proof connection from the sampling head to the pump
- Calibrated timepiece, to measure exact sampling time
- Sample record sheets (Appendix 1)

In order to achieve as near total –NCO as possible, samples of the bulk products being applied should be collected where it is likely that the available calibration standards do not reflect >80% of the NCO within the product. During analysis, these bulks should be used to estimate the total –NCO present and oligomer standards as well as monomer standards should be used for quantitation[2].

4 Recommendations and precautions for air sampling

- Users of this SOP should first carry out a suitable, specific risk assessment, prior to performing air measurements. Appropriate health and safety practices should be established in order to ensure compliance with regulatory requirements.
- Sampling is preferably carried out by a person, familiar with collecting personal inhalation measurements, according to good occupational hygiene practices
- When collecting air samples, care should be taken to avoid cross contamination of samples.
- Air sampling should be performed during a time period, which reflects the diisocyanate activity being undertaken by the exposed person.
- For short activities (15 minutes), a higher flow rate should be used (850 ml/min).
- In the event of the activity spanning a significant proportion of the work shift, it is recommended that the duration of the measurement period does not exceed 4 hours. Air sampling can be interrupted during lunch breaks if the researcher is confident that no exposure is likely. In this instance the pumps should be switched off and removed from the wearer, being replaced once the lunch break is finished. Pre-lunch and post-lunch sampling periods should be recorded in this case (see Appendix 1).
- Spare pumps, samplers and other necessary materials must always be provided so that planned samplings are not compromised.

---

[2] https://www.sigmaaldrich.com/analytical-chromatography/analytical-products.html?TablePage=17856609

5 Air sampling procedure

Michel and Schultz (2012) provides information on the sampling procedure. In addition a video is available which introduces the dry sampler and explains the sampling process etc. It is recommended that this is viewed in conjunction with reading this section https://www.sigmaaldrich.com/video/analytical/asset-isocyanates-sampler.html.

Before sampling

- Charge the pump overnight with appropriate battery charger.
- Set up the pump, sampler and flow calibrator in a clean area.
- Use powder-free gloves.
- Connect Asset sampler to be used as calibrator to the pump using flexible tubing and connectors to ensure that there is a leak proof connection from the sampler to the pump.
- This tubing should be of sufficient length allowing unimpeded movements of the worker.
- Switch on the pump and allow the flow to stabilise for a few minutes.
- Attach flow meter to the inlet of the sampler, using the calibration fitting (supplied with each pack of samplers).
- Set the flow rate at 250 ml/min for up to 4 hours or 850 ml/min for short-duration activities (+/- 5%), using a calibrated flow meter.
- Measure and record the flow rate.
- Disconnect the flow meter.
- Replace calibrator Asset sampler with that to be used for the sampling period. Switch on the pump and perform a leak test by covering the sampler's inlet or kinking the tubing.
- If the pump does not stall, this could indicate a leak and should be rectified and procedure above repeated.
- Switch off the pump.
- Recap sampler to prevent contamination of the filter.

Placement of samplers on participants

- Attach pump(s) to the worker's belt or harnesses so that they cause minimum inconvenience to the worker and safely secure the pump tubings with clips so that they do not present a safety risk to the wearer.
- Attach sampler to the worker's upper chest or lapel using clips. Samplers should be placed in the breathing zone, not more than 30 cm away from the nose-mouth region.
- When ready to begin sampling, remove protective cap from the sampler.
- Switch on the pump and record the time, using a calibrated timepiece.
- Check the sampler and pump periodically during sampling to ensure that the equipment is still working.
- In the event of the activity spanning a significant proportion of the work shift, it is recommended that the sampling does not exceed 4 hours and it should be interrupted during lunch breaks, if confident that there is no exposure. Pumps should be switched off and removed from the worker during this time. If possible, flow rates should be checked during lunch breaks and at least at 2 hourly intervals and the required flow maintained. Where flow adjustment is necessary this should be

recorded (time of adjustment and flow rate before and after adjustment) so that an accurate total air sampling volume can be calculated. Record pre-lunch and post-lunch sampling periods if necessary.

At end of sampling period

- Measure and record the flow rate before switching off the pump.
- Switch off the pumps and record the time, using a calibrated timepiece.
- Carefully disconnect the sampler from the tubing and place protective caps on either end.
- Calculate the sampled air volume by multiplying the average flow rate with the sampling duration. For example,

  08:30 sampler placed on worker and started, flow rate = 250 ml/min
  09:45 flow rate check rate, flow = 240 ml/min (flow rate reset to 250 ml/min)
  12:30 Sampler removed at lunch break and end of sampling period (flow rate = 250 ml/min)

  Sample period 1; 08:30 – 09:45 (75 mins); average flow rate = (250+240)/2 = 245
  Sample period 2; 09:45 – 12:30 (165 mins) average flow rate = (250+250)/2 = 250

  Total sample volume = (75 x 245) + (165 x 250) = 59625 ml = 59.6 litres.

- For each air sample, a sample record sheet should be completed (Appendix 1). Following items are recorded : a unique identification code (including country ID, participant ID and sample ID, as explained below), sampling date, pump ID, start and end time (of which there will be two for that sample is stopped for a lunch break), overall sampling duration (min), flow rate (mL/min) both before and at the end of each sampling duration, sampled air volume (L) and other relevant sampling information (location, activities).

8hr TWA for the workers will be calculated at the data analysis stage, using the information provided in the sample record form. It is therefore important that this is fully completed.

6 Sample traceability and contextual information

A standardised convention will be used to assign unique identification codes for all samples collected. The identification code convention is as follows:

Diisocyanate (D) - Country ID (XX) - Company ID (XX) - Participant ID (XXX) - Sample ID (AX/BX/LCX/RCX/BAX/EX/UX/WX)

'D' is to denote that the samples and data relate to the diisocyanate occupational study.

Country ID 'XX' is the country code, using the ISO Alpha-2 country codes for the participating countries (http://www.nationsonline.org/oneworld/country_code_list.htm).

Country	ISO Alpha-2 country codes
Belgium	BE
Finland	FI
France	FR
The Netherlands	NL
United Kingdom	UK

Company ID ‘XX’ is a two-digit running number of companies in each country (e.g. 01 for the first company recruited, 02 the second and so forth).

Participant ID 'XXX' is a three-digit running number of participants in each country (e.g. 001 for the first participant recruited, 002 the second and so forth).

Sample ID ‘AX’ where A denotes the type of sample collected (air), followed by an one-digit identifier (X) to identify the running number of each type of sample for that worker (e.g. 1 for the first sample, 2 for the second and so forth).

The following scenario is provided to illustrate the application of this convention. A worker is recruited in UK. He is working in the first company recruited. He is the first worker recruited in that company and is providing a first air sample. The sample identification code assigned is therefore D-UK-01-001-A1.

7 Storage of collected air samples

After sampling, air samples and associated bulk samples can be stored at ambient conditions in sealed containers free from potential contamination. Details of the time period for which the samples are stored before transportation to the lab, should be held. It is reported that samples are stable for up to 4 weeks after sampling. Samples should therefore be transported and analysed within this time frame.

8 Quality control

To check for possible contamination during the sampling procedure, transportation and storage, field blanks should be collected for each sampling survey. Submit at least one blank sample for every daily series of 1-10 samples. This blank should be handled in exactly the same way as the sampled media but with no air drawn, remembering to ensure that the protective cap is removed and replaced.

9 Transportation of air samples to laboratory

After sampling, the labelled samples, accompanied by the sample record sheets, should be transported to the agreed analytical laboratory. Ambient temperature storage conditions are acceptable.

Sample shipment should be done as soon as possible in order to ensure that the samples are analysed within 4 weeks after sampling. Details of the numbers of samples being sent, sample identification codes, requested analysis and contact details of the responsible researcher should accompany the samples.

It is recommended that a hard copy of this information be included with the samples and that an electronic version is issued to the receiving laboratory at the time of sending the samples. This will allow sample numbers and identification codes to be checked upon receipt at the laboratory.

10 Data reporting

It is important that information entered into the sample record sheet (Appendix 1) is recorded as per the instructions given in this SOP as this information will need to be entered into the data template for the overall diisocyanate study.

With respect to the air samples results, the following information should be obtained from the laboratory undertaking the analysis which is to be entered into the template:

- Air concentration (this must be in µg)
    - If concentration is below the limit of quantification (LOQ), the result is replaced by **<**LOQ (for example, <0.23 if 0.23 is the LOQ). Data below LOQ should not be given as an empty cell, zero concentration or free text (i.e. <LOQ, not detected, n.d., LOQ/2)
      - please ensure that the **<** is used to identify the result as <LOQ.
- Air volume (this must be in m^3)
- Analytical method used
- LOQ of the analytical method (this must be in µg)
- Method to calculate the LOQ (the laboratory should test that the reported LOQ concentration can be analysed accurately (RSD <20 %))

Care must be taken to follow the instructions accompanying the data template. The template must NOT be modified in any way - DO NOT add / remove columns, or alter the drop down lists, or merge cells. In the event that the template is modified or data has been provided which does not follow the instructions, templates will be returned to the data provider for correction.

References

Brown J, Barrey E, Shimelis O, Schultz K. Analysis of Isocyanates Using the ASSET™ EZ4-NCO Dry Sampler. Reporter US, 30(3) https://www.sigmaaldrich.com/technical-documents/articles/analytical/environmental/asset_ez4-nco-isocyanates.html (last accessed 27th January 2021).

ISO 17734-1:2013, Determination of organonitrogen compounds in air using liquid chromatography and mass spectrometry – Part 1 : Isocyanates using dibutylamine derivatives.

Merck KGaA, (2020) ASSET™ EZ4 Dry Samplers for Isocyanates. First Class Sampling and Analysis. https://www.sigmaaldrich.com/content/dam/sigma-aldrich/docs/Sigma-Aldrich/Brochure/1/30191-asset-ez4-brochure-mk.pdf (last accessed 27th January 2021).

Michel F and Schultz K. (2012). ASSET™ EZ4-NCO Dry sampler for isocyanate measurements in air. Available from: https://www.sigmaaldrich.com/content/dam/sigma-aldrich/docs/Supelco/General_Information/1/06_Isocyanate%20Sampling.pdf (last accessed 27th January 2021).

Porras S, Ladeira C, Ribeiro E, Viegas S, Uuksulainen S, Santonen T, Galea K, Cherrie J, Louro H, Ventura C, Silva MJ, Leese E, Jones K, Hanser O, Ndaw S, Robert A, Duca R-C, Poels K, Godderis L, Kiilunen M, Norppa H, Veijalainen H, Parshintsev E, Tuomi T, Ruggieri F, Alimonti A, Koch H, Bousoumah R, Antoine G, Jacoby N, Musgrove D. (2019) HBM4EU occupational biomonitoring study on hexavalent chromium and other harmful chemicals. Standard Operating Procedures (SOPs). WP8 - Targeted field work surveys and alignment at EU level. Available from URL: https://www.hbm4eu.eu/online-library/ (last accessed 27th January 2021).

Appendix 1: Sample record sheet

**Sample code:**		**Pump ID:**	
**Sampling date:**		**Company Name:**	
**Shipping date :**		**Company ID:**	
**Researcher:**		**Participant Name:**	
**Organisation:**		**Participant ID:**	
**Diisocyanates used during sampling period*:**			
**Activity sampled (free text description of activity sampled):**			
**Duration of activity sampled (mins)**		**No. instances worker undertook this activity during work shift (inc. activity sampled):**	
**If further activities were undertaken, were these similar in nature to what monitored?**	Yes / No<br>(free text description)		
**Bulk sample collected?**	Yes / No	**Bulk sample code:**	
**Duration of worker breaks during shift (mins)**		**Were samples left on and running during worker breaks? If so, what was the duration of this sampled break time**	Yes / No<br>......................... mins

*** collect sufficient information to also allow SDS access (e.g. manufacturer's product name and reference number)**

	Start time (00:00)	End time (00:00)	Sampling duration (min)	Flow rate (mL/min) *before*	Flow rate (mL/min) *after*	Average flow rate (mL/min)	Air volume (L)
**Period 1**							
**Period 2 (if required)**							
		**Overall sample duration:**			**Overall average flow rate:**		

Annex 7

SOP 7:

Procedure for the collection of Exhaled Nitric Oxide (FeNO) Samples

WP8

Task 8.5

Table of Contents

Table of Contents ... 1
Authors and Acknowledgements ... 2
1 Introduction ... 2
2 Exhaled Nitric Oxide (FeNO) ... 3
2.1 FeNO Collection Devices ... 3
2.2 Principles of measurement ... 3
2.3 Collection Requirements ... 4
2.3.1 Calibration ... 4
2.3.2 Quality Control ... 4
2.3.3 Equipment and Material Required for FeNO Sampling ... 6
2.3.4 Collection ... 7
2.4 Sample traceability ... 9
2.5 Data reporting ... 10
References ... 10
Appendix 1: FeNO Site visit work sheet ... 11

Authors and Acknowledgements

Lead authors:

This document has been developed by Dr Elizabeth Leese, Kate Jones and Jade Sumner from the Health & Safety Laboratory (HSL), United Kingdom.

Contributions were received from:

Karen Galea (IOM, UK), Sophie Ndaw and Radia Bousoumah (INRS, France).

This document has been created for the HBM4EU project. HBM4EU has received funding from the European Union's Horizon 2020 research and innovation programme under grant agreement No 733032.

1 Introduction

Diisocyanate exposures are known to induce respiratory health effects such as respiratory tract sensitization and asthma. There is also concern of the potential genotoxicity and carcinogenicity of diisocyanates.

In established biomonitoring, exposure to diisocyanates can be determined by measuring their human metabolites in a urine sample. However, health effects such as occupational asthma are difficult to study.

In light of this several specific health effect markers to assess airway inflammation associated with respiratory sensitisation and asthma are to be investigated, namely, diisocyanate specific immunoglobin E (IgE), inflammatory markers in blood and exhaled nitric oxide (NO) in expired human breath (FeNO). This SOP covers the sample collection and measurement of FeNO levels.

2 Exhaled Nitric Oxide (FeNO)

Nitric oxide is produced by the lungs and is present in the exhaled breath of all humans. It is the most studied exhaled biomarker, with irregularities in FeNO related to several lung diseases, particularly asthma.[1] The American Thoracic Society recommends the use of FeNO to support the diagnosis of asthma, continued monitoring of respiratory inflammation and its response to anti-inflammatory therapy.[2]

FeNO is the fractional concentration of exhaled nitric oxide in the gas phase of exhaled air. The collection of FeNO samples is a non-invasive technique, simple to perform and causes no ill health effects to people with existing respiratory conditions.

The collection and analysis of FeNO samples in this project is to further the understanding of diisocyanate exposures and their effects on workers and to explore FeNO's potential as a health effect biomarker.

2.1 FeNO Collection Devices

There are several techniques for the detection of FeNO such as chemiluminescence, electrochemical sensors and laser-based technology.[3]

Electrochemical detection is the technique favoured for this project primarily based on its low cost, light weight and hand-held portable units which allow for ease of use in the site visits.[4] The basis of electrochemical detection is the conversion of gas into electrical signals which are detected by a sensor.

There are several commercially available electrochemical sensor devices. To enable standardisation amongst the different teams collecting FeNO in this project the NIOX VERO (www.niox.com) has been chosen for the collection and measurement of samples.

The NIOX VERO (see Figure 1) is a small, hand held, battery powered device. It requires a 10-second exhalation of breath by an individual. The last 3 seconds of the 10-second exhalation are analysed by the electrochemical sensor to give a definitive FeON results in parts per billion (ppb).[4]

2.2 Principles of measurement

The measurement principle is based on guidelines established by both the American Thoracic Society and the European Respiratory Society.[5] The last three second fraction of a 10 second exhalation is evaluated for average NO concentration. The exhalation flow is controlled to 50 mL/s ±5 mL/s at an applied pressure of 10 to 20 cm H_2O. The inhaled air is NO free. NO is measured using electrochemical detection. There is a gas inlet chamber with an electrolyte (sulfuric acid solution) and hardware. The NO molecules diffuse through the membrane and reach the electrolyte. A chemical reaction takes place where one electron for each NO molecule is generated. The current is proportional to the number of converted NO molecules.

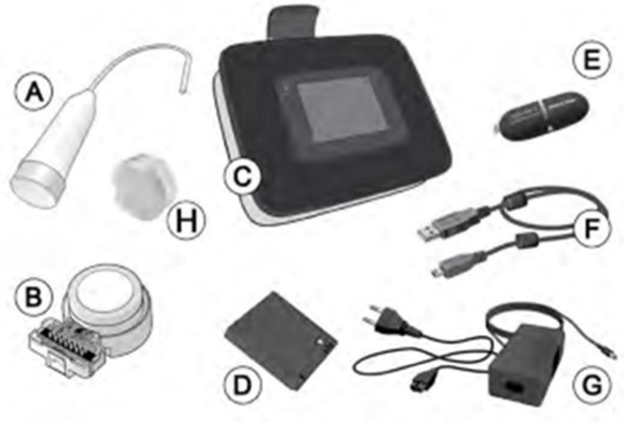

A - Breathing handle and handle cap
B - Sensor
C - Instrument
D - Rechargable battery
E - NIOX panel USB stick
F - USB cable
G - Power adapter
H - Patient filter

Figure 1. NIOX VERO instrument, accessories and parts

2.3 Collection Requirements

2.3.1 Calibration

A NIOX VERO sensor is an electrochemical sensor pre-calibrated and pre-programmed for a defined number of tests (60, 100, 300, 500, or 1000 tests). The user exchanges the sensor upon expiration (12-month lifespan). The instrument prompts the user for upcoming exchange prior to sensor expiration and does not allow for measurements with an expired sensor. Therefore, order the appropriate number of tests likely to be conducted within a 12-month period.

No additional calibration is needed during the lifetime of the sensor.

2.3.2 Quality Control

A Quality Control (QA) program is required for the user to verify the reliability of measurements. It consists of two elements; a positive biological control from a researcher with a stable FeNO value and a negative control consisting of a NO free gas sample automatically generated from ambient air.

In the 14 days prior to a site visit, baseline readings for positive and negative controls should be established by a researcher who will conduct the site visit.

In the U.S. this QA procedure is compulsory and so is incorporated into the NIOX VERO software (please see the External QC manual). You may choose to enable this (follow the QC manual) and use the device to record the control procedure, or you can do this manually and record the information on the FeNO site visit work sheet (Appendix 1).

IMPORTANT: Within the breathing handle is a NO scrubber. The purpose of the NO scrubber is to remove NO contributions from the ambient air in a workers sample. When the worker inhales through the breathing handle the scrubber removes all NO, so when the worker exhales through the breathing handle, only the sample from the workers lung airways are measured.

The expiry of the breathing handle and scrubber is 1000 tests or 1 year (whichever comes first). To ensure the NO scrubber is working correctly make sure to correctly replace the breathing handle when it expires, and to clean the device according to the manufacturers cleaning guidelines. It is also important that the handle cap be placed immediately back on the breathing handle between and after each measurement.

2.3.2.1 Negative Quality Control

The process for the negative ambient air quality control consists of performing 3 quality control (QC) measurements (ambient air) on 3 separate days within 14 days of the site visit. To establish a baseline, a mean is calculated from the 3 measurements that must be between 5-40 ppb. The mean is given a 30-day expiration date. On the day of the site visit, when on site, an ambient air measurement is taken which must be within +/- 10 ppb of the calculated mean. If this is correct then the QC has passed and the instrument is ready for use.

This single site visit QC can be performed for all site visits within the 30-day expiration date, after which the device’s negative control must be requalified by repeating the three measurements.

To measure the ambient air, attach a patient filter to the breathing handle until it clicks into place. Select the **Settings** button on the main menu, followed by the **ambient measurement** button and click the **start measurement** button. The progress bar is visible until the measurement is finished and the ambient measurement value (in ppb) is displayed. Record the value on the FeNO site visit sheet (Appendix 1)

2.3.2.2 Positive Quality Control

A researcher collecting FeNO samples on site (ideally non-smoker, no ongoing cold or airway diseases, no allergies or asthma) performs the positive control, to ensure the instrument is working accurately and to which a field positive control can be compared.

The researcher will qualify themselves and the device by performing three FeNO measurements on separate days within a 14-day period prior to the site visit. A mean is calculated from the 3 measurements that must be between 5–40 ppb. The mean is given a 30-day expiration date. To qualify the device for the day, at the site visit, the qualified user measures their FeNO which must be within +/- 10 ppb from the calculated mean. If this is correct and the negative control approved the QC has passed and the instrument is working accurately and ready to use. If it fails, the device will give an alert as to why it has failed, and suggestions to correct it.

This single site visit QC can be performed for all site visits within the 30-day expiration date, after which the user will have to requalify themselves by repeating the three measurements.

For the positive biological control, the testing procedure for giving an FeNO sample (section 2.3.4) should be followed.

2.3.3 Equipment and Material Required for FeNO Sampling

IMPORTANT: Please observe the manufacturers instruction manual for familiarisation of the NIOX VERO and for the initial setup of the instrument from new or a new sensor. Information can also be found on the website (https://www.niox.com), there are instructional videos available to aid the user manual https://www.niox.com/en-gb/professional-resources/instructional-videos/.

The NIOX VERO has the capability of being able to input worker name/ID's into the device for each sample provided, which can then be downloaded to a USB stick (please follow the manufacturer's instructions on this process). Alternatively, each sample result and remarks can be recorded on the FeNO site visit work sheet found in Appendix 1, and not stored on the device.

- Only the NIOX VERO and its individual participant filter kits can be used to collect FeNO samples. The participant filter is single use, and so a new one is required for each individual.
- A suitable room away from the primary site of exposure/workshop floor (for example, office, meeting room, first aid/nurses room) and a table and chair. For the comfort of the volunteer providing the FeNO sample, a seated position is the most suitable.
- Nitrile disposable gloves or other suitable gloves
- Hazardous waste bag for disposal of used disposable filters and gloves.
- USB stick and/or FeNo site visit work sheet (Appendix 1) and pen to record information.

2.3.3.1 General care of equipment

- DO NOT clean the instrument or handle with products containing alcohol. This includes sprays or wipes containing alcohol. Only clean the instrument and handle with a cloth dampened with water or a mild soap solution.
- Mobile phones, cordless phones or anything emitting a radio-frequency signal or electro-magnetic signal might interfere with the instrument and could make it impossible to perform a measurement so keep these out of the sampling room. Put mobile phones in aeroplane mode or turn off.
- The instrument might produce some heat during normal operation. The temperature could increase up to 5°C above the ambient temperature. Make sure that the ventilation slots are not blocked.
- Exhaled breath contains water vapor which can condense inside the instrument. When excessively used in a short period, there is a risk for condensation of water inside NIOX VERO. Normally a maximum of 10 exhalations/hour can be performed during continuous use. However, it is possible to perform 20 exhalations in one hour if the instrument is paused for a minimum of 30 minutes prior to the next session of exhalations.
- Ensure stable operating conditions by avoiding placement of the instrument in direct sunlight, near sources radiating heat, or ventilation.
- The equipment operates within specification at the following conditions: The NIOX VERO self-checks these conditions and will alert the user if not in range.
    - NO in ambient air up to 300 ppb
    - Temperature range of +10°C to +35°C

  - A relative humidity range of 20% to 80%, non-condensing
  - An atmospheric pressure range of 700 hPa to 1060 hPa
- Performance shall be sustained when measuring continuously at a pace of up to 10 measurements / hour.

2.3.4 Collection

IMPORTANT: The participant should ideally not smoke, eat or drink in the hour before the FeNO sample taken. Please advise all workers to avoid smoking, eating or drinking prior to providing their samples. This will be difficult to achieve on site, so on the FeNO site visit work sheet (Appendix 1) please record the time of testing, and whether the worker had eaten, drank or smoked within the last hour. The actual impact on results is expected to be low (1-2 ppb) but may obscure small changes.

- FeNO samples will be collected from workers in line with the urine sampling. FeNO samples are collected three times (1) before the work shift (pre-shift), (2) at the end of the work shift (post-shift), and (3) the next day in the morning. For controls, one sample is collected towards the end of the work shift (when a urine sample is also collected).
- A suitable room to collect FeNO samples should already have been decided away from the primary site of exposure/workshop floor, for example office, meeting room or nurse/first aid room. To standardise the environmental conditions as much as possible ensure the room is within general office space conditions, for example a room temperature between 18-25˚C.
- Remind the worker / research team to turn off their mobile phones or place in aeroplane mode.
- Wearing gloves, remove the handle cap from the breathing handle. Unwrap the individual patient filter and attach to the NIOX VERO breathing handle by twisting the filter into place until it clicks.
  - The filters are individually wrapped and sealed; **do not touch the filter membrane**.
  - Filters should be used immediately after opening; any already opened kits should be discarded.
- Give the breathing handle to the worker
- Ask the worker to firstly empty their lungs by breathing out thoroughly
- Ask the worker to place and close their lips around the mouthpiece of the filter of the breathing handle creating a seal so there is no leakage of air.
- Ask the worker to inhale deeply through the filter to total lung capacity (during inhalation the white cloud on the display screen will move upwards, see Figure 2).
  - Note: The activation of the display screen and white cloud should be triggered by the worker inhaling air from the handle, if this does not happen, you can press the **start measurement** button.

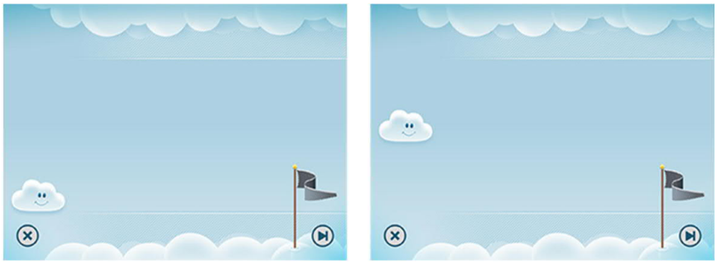

Figure 2. Example images of display screen showing cloud rising as worker inhales

- Ask them to now exhale slowly through the filter until the white cloud on the display screen passes the flag, keeping it within the white lines (see Figure 3).

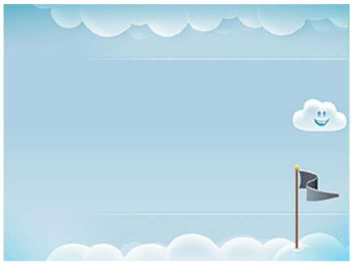

Figure 3. Example image of display screen showing white cloud passing the flag as the worker completes exhalation

  - The instrument display screen and audio signals will guide the worker to the correct exhalation pressure (see Figure 4). A continuous sound will indicate the correct pressure. The frequency of this sound will be proportional to the pressure.
  - Ask the worker to try and keep the white cloud on the display screen within the white lines as they exhale, to increase or decrease the force of their exhalation as necessary to achieve this and inform them of the different audio signals as a guide.

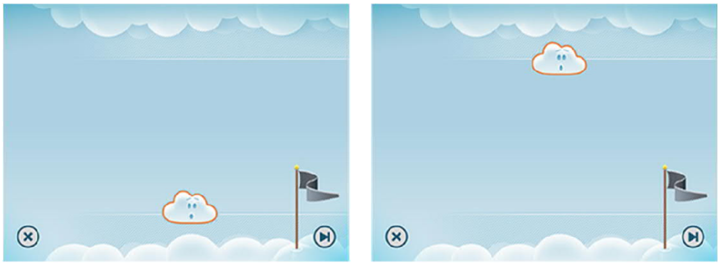

Figure 4. Example image of display screen showing exhalation being too weak or too strong

- The worker can now remove their mouth and lips from the filter.
- The instrument will now analyse the sample and generate a result, this takes approximately 60 seconds, a progress bar will be visible (see Figure 5)
  - The FeNO value is displayed on the display screen in ppb (parts per billion).

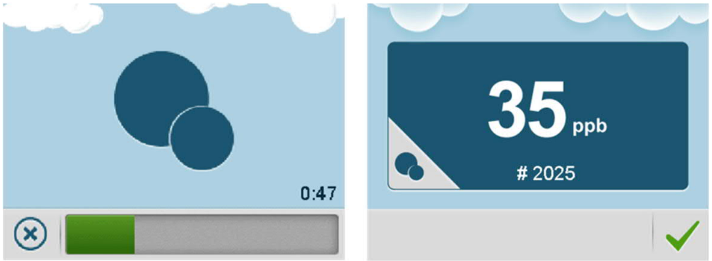

Figure 5. Example image from display screen showing progress bar of result generation and an result in ppb

- The results can either be recorded and stored on the instrument and transferred via USB stick (please see the manufacturer's instructions) or recorded on the FeNO site visit work sheet found in Appendix 1. Either worker name of worker ID can be inputted into the device. Where names are inputted into the device for identification on the instrument (for better sample management on site) these should be edited and replaced with coding (or the data deleted from the instrument) as soon as viable on return from the site visit.
- Discard the patient filter and put the handle cap back on the breathing handle.

Full details can be found in the user manual:

https://www.niox.com/pdf/niox-vero-user-manual-en.pdf.

2.4 Sample traceability

A standardised convention will be used to assign unique identification codes for all samples collected. The identification code convention is as follows:

Diisocyanate (D) - Country ID (XX) - Company ID (XX) - Participant ID (XXX) - Sample ID (AX/BX/LCX/RCX/BAX/EX/UX/WX)

'D' is to denote that the samples and data relate to the diisocyanate occupational study.

Country ID 'XX' is the country code, using the ISO Alpha-2 country codes for the participating countries (http://www.nationsonline.org/oneworld/country_code_list.htm).

Country	ISO Alpha-2 country codes
Belgium	BE
Finland	FI
France	FR
The Netherlands	NL
United Kingdom	UK

Company ID 'XX' is a two-digit running number of companies in each country (e.g. 01 for the first company recruited, 02 the second and so forth).

Participant ID 'XXX' is a three-digit running number of participants in each country (e.g. 001 for the first participant recruited, 002 the second and so forth).

Sample ID ‘EX’ where E denotes the type of sample collected (exhaled nitric oxide), followed by an one-digit identifier (X) to identify the running number of each type of sample for that worker (e.g. 1 for the first sample, 2 for the second and so forth).

The following scenario is provided to illustrate the application of this convention. A worker is recruited in UK. He is working in the first company recruited. He is the first worker recruited in that company and is providing a first exhaled nitric oxide sample. The sample identification code assigned is therefore D-UK-01-001-E1

2.5 Data reporting

The measurement range of the NERO VIOX is 5 ppb – 300 ppb and the LOD is 5 ppb. The NERO VIOX device calculates the FeNO result in ppb. The result is reported for the study as is, in ppb on site. No calculation or manipulation is required. No laboratory analysis is required.

The following information should be obtained from the laboratory undertaking the analysis which is to be entered into the data template:

- FeNO concentration (ppb)
- If concentration is below the limit of detection (LOD), the result is replaced by **<**LOD (for example, **<5ppb** LOD). Data below LOD should not be given as an empty cell, zero concentration or free text (i.e. <LOQ, not detected, n.d., LOQ/2)
- - please ensure that the **<** is used to identify the result as <LOD.
- LOD is 5ppb
- The time since the worker ate, drank or smoked within the hour before providing the FeNO sample must also be reported in minutes.

Care must be taken to follow the instructions accompanying the data template. The template must NOT be modified in any way - DO NOT add / remove columns, or alter the drop-down lists, or merge cells. In the event that the template is modified or data has been provided which does not follow the instructions, templates will be returned to the data provider for correction.

References

1. S. A. Kharitonov and P. J. Barnes, *Am. J. Respir. Crit Care Med*, 2001, **163**, 1693-1722.
2. R. A. Dweik, P. B. Boggs, S. C. Erzurum, C. G. Irvin, M. W. Leigh, J. O. Lundberg, A.-C. Olin, A. L. Plummer, D. R. Taylor and I. Amer Thoracic Soc Comm, *Am. J. Respir. Crit. Care Med.*, 2011, **184**, 602-615.
3. M. Maniscalco, C. Vitale, A. Vatrella, A. Molino, A. Bianco and G. Mazzarella, *Medical Devices-Evidence and Research*, 2016, **9**, 151-160.
4. S. E. Harnan, P. Tappenden, M. Essat, T. Gomersall, J. Minton, R. Wong, I. Pavord, M. Everard and R. Lawson, *Health Technology Assessment*, 2015, **19**, I-330.
5. S. American Thoracic and S. European Respiratory, *American Journal of Respiratory and Critical Care Medicine*, 2005, **171**, 912-930.

Appendix 1: FeNO Site visit work sheet

Date of Site Visit:

Negative (ambient air) and biological positive control qualification.

Negative QC	1.	2.	3.	mean	Date of qualification
Positive QC	1.	2.	3.	mean	Date of qualification

On site Ambient Air Result: ……………………………….. must be within ± 10 ppb of mean

On site Positive FeNO QC Result: ……………………………. must be within ± 10 ppb of mean

**Worker**<br>**Name or ID**	**Sample**<br>Pre / Post / Pre-Shift Next Day (PND)	**Time**	**FeNo Result**<br>**(ppb)**	**Notes:** Has the worker eaten, smoked or drank within the last hour. How long ago?<br>Other remarks

**Worker**<br>**Name or ID**	**Sample**<br>Pre / Post / Pre-Shift Next Day (PND)	**Time**	**FeNo Result**<br>**(ppb)**	**Notes:** Has the worker eaten, smoked or drank within the last hour. How long ago?<br>Other remarks

HORIZON2020 Programme
Contract No. 733032 HBM4EU

Annex 8

SOP 8:

Standard operating procedure (SOP) for Buccal cells sampling including sample storage and transfer Diisocyanates and E-waste occupational studies

WP8

Task 8.5

Table of Contents

Authors and Acknowledgements 3
1 Introduction 3
2 Precautions in the pre-analytical phase 4
3 Buccal cells Sampling 4
3.1 Sampling schedule 4
3.2 Sampling material 4
3.3 Instructions for buccal cells sampling 4
4 Sample traceability and contextual information 5
5 Conservation, transport and storage of the samples 6
5.1 Processing and storage of collected buccal samples at the local laboratory 6
5.2 Transportation of the samples to the laboratory 7
5.3 Storage of the samples in the laboratory until analysis 7
6 References 7
Annex 1 – Buccal cells Sampling form 9

Authors and Acknowledgements

Lead authors

Henriqueta Louro and Maria João Silva, National Institute of Health Dr. Ricardo Jorge (INSA), Lisbon, Portugal

Contributions were received from: Julia Catálan, Kukka Aimonen, Hannu Norppa (FIOH, Finland) and Maurice von Dael (Radboudumc, the Netherlands).

The text has been partially adapted for the HBM4EU occupational chromate study based on the document SOP 3: Procedure for obtaining human samples, prepared under WP 7, Task 7.2, D 7.3.

This document has been created for the HBM4EU project. HBM4EU has received funding from the European Union’s Horizon 2020 research and innovation programme under grant agreement No 733032.

1 Introduction

Genomic Instability has been observed in workers involved in processing electronic waste (Wang et al, 2018; Li et al., 2014; Liu et al., 2009).

The buccal micronucleus assay is a minimally invasive approach for measuring DNA damage, cell proliferation, cell differentiation and cell death in exfoliated buccal cells (Bolognesi et al., 2015). It offers a great opportunity to evaluate in a clear and precise way the appearance of genetic damage whether it is present as a consequence of occupational or environmental risk, being reliable, fast, relatively simple, cheap, and minimally invasive and causes no pain (Torres-Bugarín et al., 2014). Previous studies suggested that this effect biomarker can be related to waste exposure. One work reported micronuclei and other nuclear anomalies in exfoliated buccal cells from urban solid waste collectors and recyclers in southern Brazil (Brina et al 2017), and other works indicated higher frequencies of buccal micronuclei and other nuclear abnormalities such as karyolytic and karyorrhectic cells in waste pickers women (Franco de Diana et al., 2018). More importantly, increased frequencies of micronucleus, karyolysis, and pycnosis in the exfoliated buccal cells in scavenging teenagers at Alaba International market has been related to E-waste indiscriminate disposal and primitive recycling processes, possibly due to elevated Serum Pb, Ni, Cd, and Cr Levels (Alabi et al., 2020). Thus, this effect biomarker seems to be suitable to assess local effects from e-waste exposure. On the other hand, studies on workers exposed to diisocyanates utilized in the manufacture of polyurethane foam have reported increased genotoxic effects (Lindberg et al., 2011). Both toluene diisocyanate- and 4,4′-methylenediphenyl diisocyanate- exposed workers showed increased frequencies of micronuclei in peripheral blood lymphocytes (Norppa et al., 2000; Bilan, 2004) and in buccal epithelial cells (Norppa et al,, 2000), confirming the suitability of this assay to assess diisocyanates.

This guideline is intended to be used in the framework of the Human Biomonitoring Initiative (HBM4EU). The Standard Operating Procedure (SOP) for buccal cells sampling provides the general procedure for the collection, storage and transfer of buccal cell samples to be analysed within the diisocyanate and the e-waste occupational studies.

2 Precautions in the pre-analytical phase

Although quality control measures are often absent from the pre-analytical phase, it is essential to avoid, or at least minimise, sample misidentification and possible sources of contamination. In this regard, two main groups of factors should be considered:

a) Influencing factors:

   Alcohol consumption, medication intake and smoking or diet, are influencing factors that can modify the levels of MN in buccal cells. This information will be collected through questionnaire (SOP 2) and should be taken into account when analysis the results.

b) Interfering factors

   Buccal cell samples should be immediately fixed after collection and sent to the Laboratory in fixative medium to avoid cells degradation or morphology alteration. Tubes have to be appropriately coded to avoid misidentification.

3 Buccal cells Sampling

3.1 Sampling schedule

Post-shift collection is preferred.

Buccal cell collection takes approximately 10 minutes per worker.

3.2 Sampling material

The following materials and equipment will be necessary for sampling

- Small-headed toothbrushes (2-cm head length) or cytologic brushes are preferential or you can use standardised swabs (e.g.: https://isohelix.com/products/isohelix-dna-buccal-swabs/)
- 30–50 mL polystyrene containers or test tubes labeled LC (left cheek) and RC (right cheek),2 per worker
- Saccomanno's fixative (50% alcohol which contains approximately 2% of Carbowax 1540), 20 ml per worker

3.3 Instructions for buccal cells sampling

The method presented here is described in detail by Bolognesi and Fenech (2019), and is standardized and widely used by others (Thomas and Fenech, 2011; Bolognesi et al., 2013; 2017 Thomas et al., 2009). At the end of this section, an alternative procedure is provided for samples that will be processed up to 24h after collection, without being transported.

1. For each participant prepare two 30-ml polystyrene containers or test tubes, labeled with individual code and LC (left cheek) and RC (right cheek), each containing 10 ml of Saccomanno's fixative.

2. Before buccal cell collection, the mouth of the subject should be rinsed twice thoroughly with 30 ml of water to remove excess debris.

! CAUTION Human samples should be considered as infectious and the appropriate safety precautions should be taken.

3. Gently but firmly rotate a small-headed toothbrush (2-cm head length) 10 times against the inside of the cheek wall in a circular motion starting from the middle and gradually increasing in circumference to produce an outward spiral effect.

Use a different toothbrush for each cheek.

! CAUTION It is important to remember not to revisit the mouth with the same toothbrush, so as to avoid the introduction of the fixative to the mucosal lining. Use a new toothbrush for resampling.

4. The head of the brush is then placed into the fixative container and rotated such that the cells are dislodged and released into the suspension.

The cell sampling is performed on the inside of both cheeks to maximize cell collection and to obtain an homogeneous cell suspension, avoiding unknown biases that may be caused by sampling one cheek only.

5. The sampling brush should be discarded as risk waste after sampling.

6. Tightly seal the tops of the fixative containers and cover in parafilm to prevent leakage during transit from the remote collection location to the laboratory.

7. The containers are then returned to the laboratory for analysis by a courier service, and the laboratory should be informed of their shipment and anticipated arrival date, so that they can be processed as soon as possible after receipt.

8. Buccal cell suspension fixed in Saccomanno's solution can be stored at 4º C for months, but need to be washed with buccal buffer and centrifuged one or two times to be rehydrated before proceeding with next steps.

Alternative procedure:

In step 1, use Buccal cell buffer instead of Saccomanno's fixative for collecting the cells.

Buccal cell buffer preparation: 1.6 g of Tris–HCl (0.01 M), 37.2 g of ethylenediaminetetraacetic acid (EDTA) tetra sodium salt (0.1 M), and 1.2 g of NaCl (0.02 M). Weigh and dissolve in 600 mL of Milli-Q water. Make up the volume to 1000 mL. Adjust pH to 7.0 using 5 M HCl and autoclave at 121 ºC for 30 min. The buffer will last for up to 3 months when stored at room temperature.

Proceed with buccal cells washes and cell spreading within 24h (Thomas et al., 2009).

4 Sample traceability and contextual information

A standardised convention will be used to assign unique identification codes for all samples collected. The identification code convention is as follows:

E-waste (E) / Diisocyanate (D) - Country ID (XX) - Company ID (XX) - Participant ID (XXX) - Sample ID (LCX/RCX)

'E' is to denote that the samples and data relate to the e-waste occupational study.

'D' is to denote that the samples and data relate to the diisocyanate occupational study.

Country ID 'XX' is the country code, using the ISO Alpha-2 country codes for the participating countries (http://www.nationsonline.org/oneworld/country_code_list.htm).

Country	ISO Alpha-2 country codes
Belgium	BE
Finland	FI
France	FR
Germany	DE
Latvia	LV
Luxembourg	LU
Poland	PL
Portugal	PT
The Netherlands	NL
United Kingdom	UK

Company ID 'XX' is a two-digit running number of companies in each country (e.g. 01 for the first company recruited, 02 the second and so forth).

Participant ID 'XXX' is a three-digit running number of participants in each country (e.g. 001 for the first participant recruited, 002 the second and so forth).

Sample ID 'LCX' where LC denotes the type of sample collected (buccal cells, left cheek), followed by an one-digit identifier (X) to identify the running number of each type of sample for that worker (e.g. 1 for the first sample, 2 for the second and so forth).

The following scenario is provided to illustrate the application of this convention. A worker is recruited in Portugal. He is working in the first company recruited for the E-waste study. He is the first worker recruited in that company and is providing first two samples of buccal cells. One sample from the left cheek (LC) and one from the right cheek (RC). The sample identification codes assigned are therefore:

E-PT-01-001-LC1

E-PT-01-001-RC1

5 Conservation, transport and storage of the samples

5.1 Processing and storage of collected buccal samples at the local laboratory

Buccal cells fixed in Saccomanno's solution can be stored at 4ºC, allowing preservation of the cell suspensions at 4 ºC for months before processing (Bolognesi and Fenech, 2019).

5.2 Transportation of the samples to the laboratory

As a general rule, samples should be shipped to the laboratory as soon as possible. During transportation, the storage conditions precluded above should be maintained.

To **ensure samples transportation at +4°C** (max +10°C) ice packs shall be used, placed at the bottom and along the sides of the styrofoam box, making sure, however, that the samples will not freeze.

A shipping date should be arranged between the sample collectors and the laboratory. When arrangements have been finalized, the addressee should be informed of the time and means of transportation.

The deliverable report *D.7.2 "Strategy and SOPs for human sample exchange, including ethical demands"* includes all information related to the proper conservation and transport of the samples in human biomonitoring studies as well as the conditions of storage until the chemical analysis. The recommendations there referred and included in D7.2. should be followed, namely:

- Standard operating procedure for Sample Exchange on a pan-European level to be used in the HBM4EU initiative
- Shipping Category B Biological Substances
- Pro-Forma Invoice
- Sample Transfer Protocol (Manifest)
- Data Transfer Template.

5.3 Storage of the samples in the laboratory until analysis.

Once in the laboratory that will perform the analysis, the storage conditions described in the section 4.1. should be maintained, unless other specific procedure exists in the analytical lab. In addition, the slides remaining after testing will be preserved at least up to the end of the project, unless otherwise stated by national rules.

Further procedures are described in each methodology SOP.

6 References

Alabi OA, Adeoluwa YM, Bakare AA. Elevated Serum Pb, Ni, Cd, and Cr Levels and DNA Damage in Exfoliated Buccal Cells of Teenage Scavengers at a Major Electronic Waste Dumpsite in Lagos, Nigeria. Biol Trace Elem Res. 2020;194(1):24–33. Digital object identifier (DOI): 10.1007/s12011-019-01745-z

Bilban M. Mutagenic testing of workers exposed to toluene-diisocyanates during plastics production process, Am. J. Ind. Med. 45 (2004) 468–474.

Bolognesi C, Fenech M. 2019. Micronucleus Cytome Assays in Human Lymphocytes and Buccal Cells. Methods Mol Biol. 2019;2031:147-163. DOI: 10.1007/978-1-4939-9646-9_8.

Bolognesi C, Knasmueller S, Nersesyan A, et al. Inter-laboratory consistency and variability in the buccal micronucleus cytome assay depends on biomarker scored and laboratory experience: results from the HUMNxl international inter-laboratory scoring exercise. Mutagenesis. 2017;32(2):257–266. DOI: 10.1093/mutage/gew047

Bolognesi C, Knasmueller S, Nersesyan A, Thomas P, Fenech M. The HUMNxl scoring criteria for different cell types and nuclear anomalies in the buccal micronucleus cytome assay - an update and expanded photogallery. Mutat Res. 2013;753(2):100–113. DOI: 10.1016/j.mrrev.2013.07.002

Bolognesi C, Roggieri P, Ropolo M, et al. Buccal micronucleus cytome assay: results of an intra- and inter-laboratory scoring comparison. Mutagenesis. 2015;30(4):545–555. DOI: 10.1093/mutage/gev017

Brina KR, Carvalho TS, Ardenghi PG, Basso da Silva L. Micronuclei and other nuclear anomalies in exfoliated buccal cells of urban solid waste collectors and recyclers in southern Brazil. Chemosphere. 2018;193:1058–1062. DOI: 10.1016/j.chemosphere.2017.11.119

Franco de Diana D, Segovia Abreu J, Castiglioni Serafini D, et al. Increased genetic damage found in waste picker women in a landfill in Paraguay measured by comet assay and the micronucleus test. Mutat Res Genet Toxicol Environ Mutagen. 2018;836(Pt B):19–23. DOI: 10.1016/j.mrgentox.2018.06.011

Li K, Liu S, Yang Q, et al. Genotoxic effects and serum abnormalities in residents of regions proximal to e-waste disposal facilities in Jinghai, China. Ecotoxicol Environ Saf. 2014; 105, 51–58. DOI: 10.1016/j.ecoenv.2014.03.034

Lindberg HK, Korpi A, Santonen T, Säkkinen K., Järvelä M., Tornaeus J., Ahonen N., Järventaus H., Pasanen A-L., Rosenberg C., Norppa H. Micronuclei, hemoglobin adducts and respiratory tract irritation in mice after inhalation of toluene diisocyanate (TDI) and 4,4'-methylenediphenyl diisocyanate (MDI). Mutat Res. 2011;723(1):1–10. DOI: 10.1016/j.mrgentox.2011.03.009

Liu Q, Cao J, Li KQ, et al. Chromosomal aberrations and DNA damage in human populations exposed to the processing of electronics waste. Environ Sci Pollut Res Int. 2009;16(3):329–338. DOI: 10.1007/s11356-008-0087-z

Norppa H., Bernardini S., Wikman H., Järventaus H., Rosenberg C., Bolognesi C., Hirvonen A. Cytogenetic biomarkers in occupational exposure to diisocyanates: influence of genetic polymorphisms of metabolic enzymes, Environ. Mol. Mutagen. 35 (Suppl. 31) (2000) 45.

Thomas P, Fenech M. Buccal micronucleus cytome assay. Methods Mol Biol. 2011;682:235–248. DOI: 10.1007/978-1-60327-409-8_17

Thomas P, Holland N, Bolognesi C, et al. Buccal micronucleus cytome assay. Nat Protoc. 2009;4(6):825–837. DOI: 10.1038/nprot.2009.53

Tolbert PE, Shy CM, Allen JW. Micronuclei and other nuclear anomalies in buccal smears: methods development. Mutat Res. 1992;271(1):69–77. DOI: 10.1016/0165-1161(92)90033-i

Torres-Bugarín O, Zavala-Cerna MG, Nava A, Flores-García A, Ramos-Ibarra ML. Potential uses, limitations, and basic procedures of micronuclei and nuclear abnormalities in buccal cells. Dis Markers. 2014;2014:956835. DOI: 10.1155/2014/956835.

Vidyalakshmi S, Nirmal RM, Veeravarmal V, Santhadevy A, Aravindhan R, Sumathy. Buccal Micronuclei Assay as a Tool For Biomonitoring DNA Damage in Oral Lichen Planus. J Clin Diagn Res. 2016;10(7):ZC05–ZC7. DOI: 10.7860/JCDR/2016/17074.8072

Wang Y, Sun X, Fang L, et al. Genomic instability in adult men involved in processing electronic waste in Northern China. Environ Int. 2018;117:69–81. DOI: 10.1016/j.envint.2018.04.027

Annex 1 – Buccal cells Sampling form

Confidential Data – do not send with sample

Study Identification (Diisocyantes/ E-waste): ______________________________

Worker Identification:

Country: ____

Company name and name of department: ___________________________________

Worker name: ____

Position: ____

Control? __________(Yes/No)   Exposed? __________ (Yes/No)

Date: __________________________

Code Number: _________________

-- ◄◄

Send with sample for analysis

***Questionnaire*:**

Date: _____________

Code Number: _________________

Buccal Sample:

Date: _______________ Time of sampling: ___________________________

Buccal Code Number: ____

Number of Samples collected from this individual: ______________

Notes/Observations:

____

____

____

HORIZON2020 Programme
Contract No. 733032 HBM4EU

Annex 9

SOP 9:
Standard operating procedure for comparing occupational hygiene measurements with exposure estimates generated using the Advanced REACH Tool via the TREXMO model

WP 8
Task 8.5

Table of contents

Authors and Acknowledgements 2

1 Scope 2

2 Contextual information to be collected during site visits 3

3 Generation of exposure scenarios for use in modelling exercise 3

4 Model to be used 3

5 Who should complete the modelling exercise? 3

6 The modelling exercise 4

6.1 Overview 4

6.2 Background questionnaire 4

6.3 How to use TREXMO 4

6.4 Exposure situations 4

7 Data preparation 5

References 5

Appendix 1: Contextual information proforma 6

Appendix 2: Example of exposure scenarios to be generated 10

Authors and Acknowledgements

Lead authors:

This document has been developed by Karen Galea (IOM, UK) and Susana Viegas (ESTeSL-IPL, Portugal).

Contributions were received from:

Radu-Corneliu Duca (National Health Laboratory, Luxembourg), Katrien Poels (KU Leuven University, Belgium) and Sanni Uuksulainen (FIOH, Finland).

This document has been created for the HBM4EU project. HBM4EU has received funding from the European Union's Horizon 2020 research and innovation programme under grant agreement No 733032.

1 Scope

Many different approaches can be used for estimating occupational exposure to chemical substances. More recently, and probably primarily due to regulatory influence following the introduction of the REACH regulations, the use of predictive exposure models is becoming more frequent as it is not possible for the occupational hygiene community to collect a sufficient number of exposure measurements to generate estimates for all relevant exposure scenarios (Fransman, 2017; Landberg et al., 2018).

When applying exposure assessment modelling tools, users are required to select options from a number of possible input parameters. Hence, results obtained with the tools could be affected by factors such as the professional experience and judgment of the tool user and access to an appropriate level of information.

Several Tier 1 screening models such as ECETOC-TRA, MEASE, ART 1.5 and others are recommended for use under REACH (ECHA, 2016) and were evaluated under the E-TEAM project of which the results have been reported in several papers (Lamb et al, 2015, 2017, Tischer et al, 2017, van Tongeren et al, 2017). Lamb et al (2017) reported a between-user reliability exercise where exposure estimates ranged over several orders of magnitude for the same exposure situation by different users. It was also noted that the amount of contextual information provided in the situations could have potentially affected the level of variation between users. To explore this further a standardised proforma will be used in the HBM4EU occupational studies to collect contextual information about the work activities observed during the measurement campaign. For each visit, an exposure scenario will be generated.

At a later stage (and without knowledge of the results of the measurement campaigns), participants with differing knowledge about the workplace environments and activities will be given the generated workplace exposure scenarios and asked to use a selected REACH model, ART 1.5 via the TREXMO (TRanslation of EXposure MOdels) tool with participants being asked to estimate inhalation exposure. TREXMO is a tool primarily developed to efficiently and reliably assess the wide variety of exposure situations using the available occupational exposure models. TREXMO integrates six commonly used occupational exposure models: ART v.1.5, Stoffenmanager® version 4.0, ECETOC TRA v.3, MEASE v.1.02.01, EMKG-EXPO-TOOL and EASE v.2.0, although we will only be using the outputs of ART 1.5 (the higher exposure assessment tool)**.** Comparisons of the exposure estimates generated between the different types

of users will be made, with these estimates also being compared with the actual exposure measurement results.

This Standard Operating Procedure (SOP) is partially based on earlier work undertaken by the IOM which has focused on the evaluation of exposure models (e.g. Lamb et al, 2015;2017; van Tongeren et al, 2017). This SOP is focused on: a. the collection of contextual information to inform the development of exposure scenarios to be used in the modelling exercises and b. the administration of the modelling exercise to participants. Details of how the collected data will be analysed is not provided in this SOP.

2 Contextual information to be collected during site visits

A standardised proforma will be used to gather relevant contextual information during the field survey measurement campaigns (Appendix 1). Information to be gathered will be from researchers first hand observations of the workplaces and activities taking place there and will include, for example, details of the risk management measures in place and used, operational conditions, materials generated, used etc. This proforma is to be completed on the same day that the air and other environmental samples are collected.

3 Generation of exposure scenarios for use in modelling exercise

The competed proformas will be returned to KG (karen.galea@iom-world.org) and SV (susana.viegas@ensp.unl.pt), who will use these to generate exposure scenarios to be used in the modelling exercise.

A standardised single A4 page format will be used for the generated exposure scenarios to minimise participant uncertainty from differences in layout of the descriptive information. An example of what these may look like is provided in Appendix 2 (this example being from the IOM E-TEAM project).

4 Model to be used

Participants will be asked to generate inhalation exposure estimates for the various scenarios using the higher tier REACH model, ART 1.5. This will be via the TREXMO tool, http://trexmo.chuv.ch/.

5 Who should complete the modelling exercise?

Each participating country where air samples will be collected as part of the biomonitoring campaigns, are invited to participate. In each country the following participants will be directly involved:

- The occupational hygienist / researcher who completed the contextual information template and collected the air and and other environmental samples.
- A member of the project team who did not visit the sites.
- An individual experienced in exposure assessment but who has no direct experience of the projects or sites where exposure to the chemicals being studied was assessed.

6 The modelling exercise

6.1 Overview

Participants will be asked to complete a short background questionnaire. They will then be provided electronically with a pack containing simple instructions for completing the exercise, instructions on how to use TREXMO (ART 1.5), the exposure scenarios and supporting worksheets. Details of how the assessments are to be returned will also be provided.

6.2 Background questionnaire

A short background questionnaire will be administered to collect key information on participants' experience in relation to the measurement campaign and also their use of modelling tools.

The participants will be requested to provide the following information:

- Organisation they work for.
- Years of experience in exposure assessment.
- The nature of their involvement in the measurement campaigns, e.g. if they personally collected the contextual information / air and other environmental samples at the sites; if they did not attend any site visits; had no involvement with the studies being developed.
- Previous experience with the use of the model.

6.3 How to use TREXMO

A simple guide to gaining access to TREXMO and how to use the ART 1.5 model for the purposes of the exercise will be provided. Guidance and screenshots detailing the required tool outputs will also be included.

6.4 Exposure situations

Depending on the number of exposure situations which are eventually generated, participants may receive these in batches or all at once.

For each exposure scenario, participants will be instructed to undertake an inhalation exposure assessment using ART 1.5, even where the scenario may be outwith the scope for the tool. For each exposure scenario they will be asked to complete a worksheet to record their results .

For each exposure scenario issued, participants will be required to document systematically the following contextual information on the worksheet:

- Previous experience of the given exposure scenario.
- Instances where they found choice or description of parameter types difficult, i.e. the level of uncertainty in their choice for example when selecting substance characteristics or risk management measures.
- The outputs derived by the tool.
- Their perception of the level of over/ under-estimation of the exposure estimate generated by the tool.

Participants will be asked to complete the given exposure scenario and return the completed worksheet within a specified period of time. A reminder will be issued in the event of non-receipt.

7 Data preparation

The exposure assessment outputs will be harvested from the returned worksheets and questionnaires and tabulated for analysis in Microsoft Excel spreadsheets.

References

ECHA Guidance Part D. European Chemical Agency, 2016. In: Guidance on Information Requirements and Chemical Safety Assessment, Chapter R.14, version 3.0, Helsinki, Finland.

Fransman, W. (2017) How accurate and reliable are exposure models? Annals of Work Exposures and Health; 61(8): 907-910. https://doi.org/10.1093/annweh/wxx068

Landberg, H.E., Westberg, H., Tinnerberg, H. 2018.Evaluation of risk assessment approaches of occupational chemical exposures based on models in comparison with measurements. Safety Science, 109: 412–420

Lamb, J., Galea, K.S., Miller, B.G., Spankie, S., Van Tongeren, M., Hazelwood, G., 2015. Evaluation of Tier 1 Exposure Assessment Models under REACH (eteam) Project – Substudy report on between-user reliability exercise (BURE) and workshop. Dortmund. Avaliable from. https://www.baua.de/EN/Service/Publications/Report/F2303-D22.pdf?__blob=publicationFile&v=2

Lamb, J., Galea, K.S., Miller, B.G., Hesse, S., Van Tongeren, M. 2017. Between-User Reliability of Tier 1 Exposure Assessment Tools Used Under REACH. Annals of Work Exposures and Health,61(8):939-53. https://doi.org/10.1093/annweh/wxx074

Tischer, M., Lamb, J., Hesse, S., van Tongeren, M. 2017. Evaluation of Tier One Exposure Assessment Models (ETEAM): Project Overview and Methods. Annals of Work Exposures and Health, 61 (8): 911–920. https://doi.org/10.1093/annweh/wxx066

Van Tongeren, M., Lamb, J., Cherrie, J.W., Maccalman, L., Basinas, I., Hesse, S. 2017. Validation of lower tier exposure tools used for REACH: comparison of tools estimates with available exposure measurements. Annals of Work Exposures and Health. 61(8):921–938. https://doi.org/10.1093/annweh/wxx056

Appendix 1: Contextual information proforma

**Worker Information:**	
Date:	
Participant ID:	
Job Title:	
Shift Length (hours)	

**General working environment**	
Location	Indoor / Outdoor
Room temperature	°C
Approximate size of room where the participant works	m3
Ventilation in room	Natural / Mechanical / Both
Air change rate	air changes per hour
Free text description of how well room is ventilated	
General impression of cleanliness and tidiness of workplace	Poor / Good / Excellent
Frequency of cleaning per day and how	

**General hygiene provision**	
Hand-washing facilities in immediate work location?	Yes / No
Do workers shower at end of the shift?	Yes / No
Separate eating/ drinking area?	Yes / No
Others:	

**Training**	
Workers received previous training on the health risks associated with their work?	Yes / No
If yes, please specify	

Activities and tasks within these	
Description of activities taking place in this environment and numbers of workers involved (for each activity and in total)	
Description of tasks undertaken by workers and how they may be exposed:	
Process classification (continuous/batch) across operations and tasks	
Level of automation, mechanisation and manual interventions in process	
Any tools being used / manipulated and how this is done?	Description of tools used :<br><br>Please shortly describe the tools utilization for the different task:<br><br>Time spent on each task / activity during the day
Approximate working distance of worker from the exposure source	……………m<br><br>If different distances for different workers please specify
Any observed differences in working behavior between workers involved in same activity?	
Were the tasks /activities	Yes / No

observed on the day typical of usual work activities?	If no, were samples/information collected during periods of lower / higher work rates?<br><br>How do these differ over the course of a week/year?

Nature and sources of hazardous substance	
Physical state of substance:	solid……………. Dustiness: ………………..<br><br>liquid……………. Vapour pressures (Pa) at 20°C: ………………....
Concentration of hazardous substances being assessed in preparation/product?	☐ <1%<br>☐ 1-5 %<br>☐ 5-25%<br>☐ >25%<br>☐ 100%
Sources of emission and subjective assessment of where emissions may be high	

Operational conditions	
Frequency and duration of exposure of workers conducting the tasks that can imply exposure (e.g. Task X: 120 min. twice a shift)	
Amount of substance handled:	Kg per shift
Use rate (include units):	
Process conditions that can be relevant (e.g. heated bath, high current applied)	
Process temperature	°C
Level of automation (e.g. manual)	

Risk management measures (description and comment on each of these	
Segregation – description	
Enclosure	
Local exhaust ventilation controls, description and comment on position, use, effectiveness	
Suppression techniques	
Control rooms description – time spent (minutes or hours)	
Others	

PPE	
RPE usage by worker during activities, cleaning/storage regime	Type of RPE used: ……………………………………..<br><br>Supplier APF: …………………<br><br>% of time/tasks being used:
Protective gloves	Type: ……………………………………..<br><br>% of time/tasks being used:

Appendix 2: Example of exposure scenarios to be generated

Example exposure situation from E-TEAM project (Lamb et al, 2017).

Situation 15: Packing of Nickel Metal Powder

Please assess inhalation and dermal exposure to **nickel** in the situation described below.

When entering data into the tools during the exercise, please use the CAS number, molecular weight and vapour pressure value given in the table below.

1. General Description of Exposure Situation

This situation describes the packing of nickel powder in drums.

The operator removes excess powder (Product R) from a pre-weighed drum using a hand scoop and places the surplus material into a storage bin located at the packing station (Work Area R). If the containers are below the required weight, the operator uses the scoop to transfer powder back from the storage bin into the drum.

The operator then fixes a sealing cap onto an open aperture on the top of the drum.

The packing station is provided with local exhaust ventilation at the filling point. An air assisted filtering visor fitted with P3 filters is worn. All packing operators wear cotton overalls and safety boots. Gloves are not worn during scooping of powders.

The activity takes place at room temperature (20ºC) in a small room with general ventilation.

The activity takes place for approximately 3 hours per 8 hour shift.

2. Product/ Substance Information

Product	Supplier	Substance Name	CAS Number	Molecular Weight/ $gmol^{-1}$	Vapour pressure at 20ºC/ Pa	Concentration of Nickel in Product R (%)
Product R	Supplier R	Nickel	7440-02-0	59	1 (Negligible)	100

HORIZON2020 Programme
Contract No. 733032 HBM4EU

Annex 10

SOP 10:

Communication plan for the occupational studies

(diisocyanates and E-waste)

WP 8

Task 8.5

Table of Contents

Authors and Acknowledgements... 2
1 General introduction... 2
2 Communication Plan.. 3

Authors and Acknowledgements

Lead authors:

This document has been developed by Susana Viegas from Escola Superior de Tecnologia da Saúde de Lisboa (ESTeSL), Instituto Politécnico de Lisboa, Portugal.

Contributions were received from: Karen Galea (IOM, UK), Bernice Schaddelee-Scholten (TNO, the Netherlands), Radia Bousoumah, Sophie Ndaw (INRS, France) and Tiina Santonen (FIOH, Finland).

This document has been created for the HBM4EU project. HBM4EU has received funding from the European Union's Horizon 2020 research and innovation programme under grant agreement No 733032.

1 General introduction

In the scope of HBM4EU project two further occupational studies will be developed.

The first is focused in diisocyanate exposure in the manufacturing and repair of large vehicles (non-booth spraying of e.g. boats/planes), the use of diisocyanate based hot-melt glues in different sectors, and construction sector, which includes different sources of diisocyanates exposure (floorings/screeds, insulation). Its main aims are to provide new data on the exposure to diisocyanates in specific sectors based on harmonized sampling protocols, test the usability of different biomarkers in the assessment of exposure to diisocyanates, and use the collected data to validate the PBPK model developed. The study will be conducted during 2020-2021 in five countries: Belgium, Finland, France, The Netherlands and United Kingdom.

The second study is dedicated to occupational exposures in the E-waste handling sector. The main aim is to contribute to awareness of potential hazards and stimulate good work practices that will lead to further improve protection of the worker's health from the risk of exposure to toxic components, including that of combined exposures. The study will include the assessment of exposure to several HBM4EU priority compounds, including metals (lead, inorganic mercury, cadmium, chromium), phthalates, and flame retardants. The study will be performed in Belgium, Finland, Germany, Hungary, Latvia, Luxembourg, The Netherlands, Poland, Portugal and United Kingdom.

This Communication Plan SOP is designed to ensure that all the relevant results of these studies are communicated to all study participants (companies, workers and equally to controls) and national and EU stakeholders. Dissemination of information to the scientific community is also covered. The results will be presented in different manner dependent of the type of participant (e.g. individual levels for workers and or aggregated levels to companies). Effective and consistent communication with participants and stakeholders will help ensure that the results obtained are accessible to support organizational learning and used for decision-making. This will happen at the companies and workers level but also at regulatory level. To sum up, it is CRUCIAL to consider that communication is very relevant and it is a way to guarantee that the tools and data being developed in the scope of HBM4EU will be available to be used by risk assessors.

This SOP has been created with the premise in mind that every participating country is obliged to follow the HBM4EU documents and guidance concerning ethical aspects and also the national ethical committees.

2 Communication Plan

Considering the above a communication plan is proposed for both occupational studies (Table 1). This communication plan describes the different target audiences, the objectives/actions desired, the message content for each stakeholder/group, the method of choice to convey the message and, finally, the best moment in time for the message to be communicated. Additionally, it is important to consider that this is a living document and it will be reviewed and revised as necessary throughout the full duration of the occupational studies.

Table 1 – Current communication plan

Stakeholder/group	Objectives/actions desired	Data to be communicated	Delivery methods/venue	When
Study participants (workers and controls)*	Fulfill conditions set in informed consent procedure.<br>Inform the workers about their individual results to support behavioral change if needed	Individual results of exposure as per study information leaflet and consent form. In some countries they will only be provided to the study persons on request. Extra care will be given to the communication of unexpected results (procedure as specified in approved study protocol).	Depending on the country, for example via occupational physician of the company, or directly by the responsible researcher/physician of the national research group. Workers are given an opportunity to discuss on their results and on their meaning.	When the results are available for each company engaged in the project.
Participating companies**	Inform companies about the exposure results to support the risk assessment, definition of priorities and improve risk management measures.	Results of the occupational hygiene (air and wipe/settled dust) samples).<br>Results of the exposure biomarkers specified in information leaflet and informed consent form will be provided in an aggregated manner guaranteeing that workers identities are not perceived; Recommendations for RMMs if needed.	Technical report be issued to the authorised person who consented to the company participating (unless otherwise specified)	When the results are available for each company engaged in the project.
HBM4EU partners	Provide study results and recommendations with respect to the outlined study aims and objectives. This intends to be a continuing process.	Exposure data obtained in each country will be combined with the other countries and statistical analysis will be performed	Deliverable report, presentations in consortium meetings and training school (lessons learned)	When the results are ready, by the due date of Deliverable report to HBM4EU.
HBM4EU partners and other interested partners	Inform about the methodology to be employed in the occupational exposure monitoring campaigns	Developed finalized SOPs	HBM4EU training school<br>Website<br>Publications<br>Linkedin	When SOPs have been finalized.
Scientific and professional community	Inform about the most suitable biomarkers for each substance, exposure levels,	Exposure data obtained in each country will be combined with the	Scientific publications in peer reviewed journals;	Begins already when the methodology has been

Stakeholder/group	Objectives/actions desired	Data to be communicated	Delivery methods/venue	When
	variables that influence exposure, occupational settings with higher exposure, RMMs with higher efficacy	other countries and statistically analysis will be performed.	presentations at conferences and seminars (at national and international level) and online webinars	established, and continues when the results are ready.
HBM4EU stakeholders (ECHA, EU-OSHA and national contact points via their focal contact point network, DG Santé, DG Employment) and other interested parties (industry associations, workers unions, national authorities, ISES-Europe and European industrial hygiene associations)	Inform about more suitable biomarkers, occupational settings with higher exposure, evaluate the impact of the regulatory actions already in place, the support of new regulatory actions if needed.	Aggregated exposure data	Articles in HBM4EU newsletter<br>Policy brief<br>HBM4EU webpages<br>Presentations in HBM4EU stakeholder forum.<br>Deliverable report<br>Webinar/seminar on the results targeted to key stakeholders.<br>Slides from the Webinar published in web.<br>Information is shared also using social media channels.	Dates to be defined.<br>Communication begins already when the studies start and continues throughout the study.
Targeted communication to ECHA Committees if relevant (e.g. RAC).	Evaluate the impact of the regulatory actions already in place, the support of new regulatory actions if needed.	Aggregated exposure data	Presentation in ECHA	When the results and deliverable report are ready
National industry stakeholders, workers unions (optional, depending on country)	Inform about the exposures in different occupations, recommendations for the assessment and management of exposure.	Aggregated exposure data	Press release in national language, description of the study published in the website of the participating institutes. Possibly short articles in national journals targeted to relevant industry fields. Use of social media to distribute information also nationally.	This is the responsibility of each participating institute (dates do be defined)

* Communication starts in the first contact companies/workers and information about why they should participate in the study and what are the hazards related to their exposures should be provided since the beginning of the study.

** Preferably done before any reporting to HBM4EU, stakeholders and scientific community.
