# Supplementary material for: HBM4EU Diisocyanates Study—Research Protocol for a Collaborative European Human Biological Monitoring Study on Occupational Exposure
Source: Int J Environ Res Public Health. 2022 Jul 20;19(14):8811. doi: 10.3390/ijerph19148811 (PMC9319997; doi:10.3390/ijerph19148811)
Supplement: Supplementary file 1 [file ijerph-19-08811-s001.zip › File S2 Questionnaires.pdf]

HORIZON2020 Programme
Contract No. 733032 HBM4EU

HBM4EU Study Questionnaire

Exposure to diisocyanates

12th January, 2021

Content:

PART A: Self-administered questionnaire for workplaces

PART B and C: Post-shift questionnaire for workers (interviewed by researcher, filled also by control workers)

PART D. Job descriptions (interviewed by researcher during the sampling)

PART E. Questionnaire on respiratory health and symptoms for workers (filled also by control workers)

A. QUESTIONNAIRE FOR WORKPLACES (self-administered)

We would be grateful if you can complete this short questionnaire concerning your companies' activities relating to di-isocyanate exposure. Please return it directly to the researcher once completed.

Company and Occupational Health care information

Name and position of the company representative: ....

Name of the Company/Organisation: ....

ID number of the company: (to be filled by researcher)

....

Name of the department: ....

Site address: ....

Country: ....

Industrial sector (to be filled by researcher):

NACE Rev.2 code (to be filled by researcher): ....

Description of the workplace (nature of the business and what is being manufactured):

....

....

Could you describe what kind of information and/or training were given to the workers about the health risks in your company concerning working with diisocyanates?

....

....

Has environmental monitoring of diisocyanates been done in your workplace?

....

....

Do the workers wear and/or use personal protective equipment when working with diisocyanates? If yes could you specify?

....

....

What kind of collective preventive measures (i.e. chemical hoods) are used in the workplace when di-isocyanates are being used?

……

……

Occupational Health care information

Do you have company's own occupational health care or are these services provided by an external service provider?

(Please circle) Own External

Name and address of the Occupational health care: ……………………………………………………………………

……

Contact details of the Occupational Health and Safety department:

Name of representative: ……………………………………………………………………

Phone number: ……………………………………………………………………

Email address: ………

Operational conditions

Job	Take place at your site? (tick if apply)	Complete questions in Sections
1. Use of diisocyanate based glues, adhesives or sealants	☐	1 (this page) and 3 (page 6)
2. Coating large surfaces (e.g. floors) with polyurethane coatings	☐	1 (this page) and 3 (page 6)
3. Spray application of urethane foam in construction or boat manufacturing sector	☐	1 (this page) and 3 (page 6)
4. Spray coating of vehicles with di-isocyanate containing paints and primers	☐	1 (this page) and 3 (page 6)
5. Welding, grinding and flame cutting of polyurethane painted materials or e.g. polyurethane-insulated district heating pipes	☐	2 (page 5) and 3 (page 6)

Section 1. Includes operational conditions of Jobs 1-4

1. Information on the products containing diisocyanates

Name of the product	Name and quantity of diisocyanates in the product (%)	Average quantity of product used per month litres (l) or gallons (gal)

2. Frequency of installation/spraying/glueing and other operations using diisocyanate containing products? (categories: daily, weekly, monthly, other, don’t know)

3. Size of the parts installed/sprayed/glued? (please describe)

4. How many employees work on these activities?

Section 2: Operational conditions of Job 5. (Welding, grinding and flame cutting of polyurethane painted materials or e.g. polyurethane-insulated heating pipes)

1. The frequency of welding/grinding/flame cutting (specify the process) operations? (categories: daily, weekly, monthly, other, don't know)

____

____

What process(es) is(are) used? (please tick box)	☐ Welding<br>If yes, what welding method is used?<br>o MMA (manual metal arc)<br>o Other (please specify) ……………………………………….<br>…………………………………………………………………………..<br>☐ Grinding<br>☐ Flame cutting

2. Size of the parts worked with? (please describe)

____

____

3. How many employees work on these activities?

____

Section 3: Previous measurements

Have any of the following types of measurements been collected from your workers at the site to assess di-isocyanate exposure?

Measurements	Tick all that apply	Years collected
Air samples	☐	
Dermal exposure measurements	☐	
Blood samples	☐	
Urine samples	☐	
Other (please specify)	☐	

Would you be willing to allow the researchers to have access to these results (in a confidential manner)?
(Please circle) Yes No

If yes, contact person and contact details (e-mail, phone number):

Thank you for filling the questionnaire!

Please return it directly to the researcher once completed.

WORKER ID............................

B. POST-SHIFT QUESTIONNAIRE FOR WORKERS (interviewed by researcher, filled also by control workers)

Background information about worker

Urine sample	Date collected:<br><br>Sample code:	Time:
FeNO	Date collected:<br><br>Sample code:	Time:
Blood sample	Date collected:<br>Sample code:	Time:
Air sample (personal)	Date collected:	Sample code:
Wipe sample(s) (personal)	Date collected:	Sample code(s):
Company code		

WORKER ID............................

Worker name and position	
Sex (please circle)	Male Female
Date of birth (dd/mm/yyyy)	

Interviewer: Name, position and contact details (e-mail, phone number)

Please separate this sheet (pages 6 and 7) from the Questionnaire

----

WORKER ID..............................

C. POST-SHIFT QUESTIONNAIRE FOR WORKERS (continues; interviewed by researcher, filled also by controls)

What is your height (cm or feet/inches)	.... cm / .... ft .... inches	
What is your current weight (kg or stones/lb)	.... kg / .... St .... lb	
Occupation	Free description:	ISCO08 code
Is the work done predominantly (please circle)	Inside Outside	
Duration of work shifts (hours)<br><br>and typical overtime per week (hours)		
Type of work shifts (please tick box)	☐ Fixed day<br>☐ Fixed night<br>☐ Rotating day/back<br>☐ Rotating day/back/night<br>☐ Other (please specify)...	
Home address		
Home location and related characteristics (please circle)	Urban Rural	
Are there industrial plants, incinerators or landfill sites in the surroundings of your house? (please circle)	Yes No<br><br>If yes, approximately how far away from your house is the closest one (km)?	
Please describe the vehicular traffic density in the surroundings of your home address (please circle)	Pedestrian road (very low density)<br>Quiet street (low density)<br>Residential road (medium density)<br>Main Road (heavy density)<br>Highway (very heavy density)	
Cigarette smoking (please circle)	Yes No Former smoker	

WORKER ID............................

Cigarette smoking (continues)	Approximate number of cigarettes/day<br>Number of years you have smoked<br>If former smoker, how many years ago did you stop smoking?<br>Approximate number of cigarettes/day you smoked<br>Number of years you smoked
Do you smoke electronic cigarettes? (please circle)	Yes No Former e-cigarette
E-cigarettes (continues)	How many times you usually recharge the tank of your e-cigarette in a day?<br>Please specify how many milliliters corresponds to each refill:<br>Number of years you have smoked e-cigarettes<br>If former e-cigarettes smoker, how many years ago did you stop smoking?<br>How many milliliters you approximately used in a day?<br>Number of years you smoked e-cigarettes
Do you use any other tobacco products? (please circle)	Yes No Former user<br>If yes or former user, please specify
Other tobacco products (continues)	Approximate number of tobacco product/day<br>Number of years you have used<br>If former user, how many years ago did you stop?<br>Approximate number of product/day you used<br>Number of years used
During the past 3 months, have you had a medical x-ray or Computerised Axial Tomography (CAT) scan? (please circle)	Yes No
Have you previously, or are you currently, being treated for cancer? (please circle)	Yes No

WORKER ID………………………..

Alcohol consumption	Yes No<br><br>How often do you typicially drink alcohol? (please circle)<br><br>daily weekly monthly<br><br>Please specify the type of alcoholic beverage (e.g. wine, beer, whiskey, etc)
Alcohol consumption (continues)	On average, how many days in a month do you have at least one alcoholic beverage?<br><br>On a typical day that you drink alcohol, how many drinks do you usually have?
Consumption of other beverages (please circle)	Coffee Tea Energy drinks<br><br>On average, how many times in a typical day?<br><br>Coffee ………. Tea ………. Energy drinks ……….
Dietary habits (please circle)	Mixed Vegetarian Vegan<br><br>Other (please specify)
Recreational activities or hobbies which may cause additional isocyanate exposure (e.g. **Machining of polyurethane containing products, Home renovation and repair work, which includes the use of adhesives and sealants, polyurethane foams, paints and coatings Motor vehicle repair (especially spray painting)**	Yes No<br><br>If yes, please specify:<br><br>Duration:

WORKER ID.............................

Occupational history including the present occupation

Occupation / job title	Did the work involve any of the following activities (tick that apply)						Start time (year)	Finish time (year)
	Use of diisocyanate based glues, adhesives or sealants	Coating large surfaces (e.g. floors) with polyurethane coatings	Spray application of urethane foam in construction or boat manufacturing sector	Spray coating of vehicles with di-isocyanate containing paints and primers	Welding, grinding or flame cutting of polyurethane-painted materials e.g. polyurethane-insulated heating pipes	Other work involving exposure to diisocyanates		
	☐	☐	☐	☐	☐	☐		
	☐	☐	☐	☐	☐	☐		
	☐	☐	☐	☐	☐	☐		
	☐	☐	☐	☐	☐	☐		

Job description

What job were you doing today?

Job	(Tick if apply)	Complete questions in Job description section
1. Use of diisocyanate based glues, adhesives or sealants	☐	Section D1, page 13
2. Coating large surfaces (e.g. floors) with polyurethane coatings	☐	Section D2, page 15
3. Spray application of urethane foam in construction or boat manufacturing sector	☐	Section D3, page 17
4. Spray coating of vehicles with di-isocyanate containing paints and primers	☐	Section D4, page 19
5. Welding of polyurethane-insulated district heating pipes	☐	Section D5, page 21

WORKER ID..............................

D. JOB DESCRIPTIONS (interviewed by researcher during the sampling)

1. Job Description: Use of diisocyanate based glues, adhesives or sealants (please list the type of work tasks you have been involved in today)

	Work task	Duration of the task in a work shift (hours/ minutes)	Frequency of the task (x times per week)	Process type (manual or automatic)	PPE* used (add correspon ding numbers)	LEV** used (yes, no)
1	Preparation tasks					
2	Application of glues					
3	Cleaning and maintenance of equipment					
4	Waste management					
5	Other (please specify)					

*PPE (Personal protective equipment) worn:
1. Powered or air-fed, filtering respirator
2. Reusable half or full face mask respirator (not powered or air-fed)
3. Disposable face mask
4. Other Respiratory Protection Equipment (please specify)
5. Coveralls
6. Reusable Gloves
7. Disposable gloves
8. Other (please specify)

** LEV=local exhaust ventilation

If used, has your half or full face mask respirator been fit tested? (please circle)	Yes No<br>If yes, when?
Have you received information, instruction or training on the use of safe work practices when carrying out this activity? (please circle)	Yes No<br><br>If yes, when?
What kind of general ventilation is used in the workplace when di-isocyanates are being used?	☐ Natural (e.g. open doors / windows)<br>☐ Mechanical (e.g. fans, blowers)<br>☐ Both<br>☐ Do not know
Hygiene facilities in the company (please tick box if apply)	☐ Possibility to wash hands<br>☐ Take shower<br>☐ Separate place for working clothes<br>☐ Specific place for the storage of respiratory

WORKER ID.............................

	protective equipment<br>☐ Other (please specify)..<br>☐ ....
Have the work conditions been normal during the work day? (please circle)	Yes No<br>If not normal, please specify (e.g. higher production levels, equipment breakdowns, etc.):

WORKER ID............................

2. Job description: Coating large surfaces (e.g. floors) with polyurethane coatings (please list the type of work tasks you have been involved in today)

	Work task	Duration of the task in a work shift (hours/ minutes)	Frequency of the task (x times per week)	PPE* used (add corresponding numbers)	LEV** used (yes, no)
1	Preparation tasks: decanting, mixing, refilling				
2	Carrying the resin to the application area				
3	Spreading the resin with trowels and smoothing with rollers				
3a	Is the spreading done standing up or on hands and knees (please circle)?	Standing<br>Kneeling			
4	Drying/self-curing				
5	Cleaning and maintenance of equipment				
6	Waste management e.g. disposal of spare product				
7	Other (please specify)				

*PPE (Personal protective equipment) worn:
1. Powered or air-fed, filtering respirator
2. Reusable half or full face mask respirator (not powered or air-fed)
3. Disposable face mask
4. Other Respiratory Protection Equipment (please specify)
5. Coveralls
6. Reusable Gloves
7. Disposable gloves
8. Other (please specify)
** LEV=local exhaust ventilation

If used, has your half or full face mask respirator been fit tested? (please circle)	Yes<br>If yes, when?	No
Have you received information, instruction or training on the use of safe work practices when carrying out this activity? (please circle)	Yes<br><br>If yes, when?	No
What kind of general ventilation is used in the workplace when di-	☐ Natural (e.g. open doors / windows)<br>☐ Mechanical (e.g. fans, blowers)<br>☐ Both	

WORKER ID............................

isocyanates are being used?	☐ Do not know
Hygiene facilities in the company (please tick box if apply)	☐ Possibility to wash hands<br>☐ Take shower<br>☐ Separate place for working clothes<br>☐ Specific place for the storage of respiratory protective equipment<br>☐ Other (please specify)..<br>☐ ....
Have the work conditions been normal during the work day? (please circle)	Yes No<br>If not normal, please specify (e.g. higher production levels, equipment breakdowns, etc.)

WORKER ID............................

3. Job description: Spray application of urethane foam in construction or boat manufacturing sector (please list the type of work tasks you have been involved in today)

	Work task	Duration of the task in a work shift (hours/ minutes)	Frequency of the task (x times per week)	PPE* used (add the correspon ding numbers)	LEV** used (yes, no)
1	Preparation tasks: decanting, mixing, refilling				
2	Manual spreading of the isocyanate-based foam insulation				
3	Drying/self-curing				
4	Cleaning and maintenance of equipment				
5	Waste management				
6	Other (please specify)				

*PPE (Personal protective equipment) worn:
1. Powered or air-fed, filtering respirator
2. Reusable half or full face mask respirator (not powered or air-fed)
3. Disposable face mask
4. Other Respiratory Protection Equipment (please specify)
5. Coveralls
6. Reusable Gloves
7. Disposable gloves
8. Other (please specify)
** LEV=local exhaust ventilation

If used, has half or full face mask respirator been fit tested? (please circle)	Yes No<br>If yes, when?
Have you received information, instruction or training on the use of safe work practices when carrying out this activity? (please circle)	Yes No<br><br>If yes, when?
What kind of general ventilation is used in the workplace when di-isocyanates are being used?	☐ Natural (e.g. open doors / windows)<br>☐ Mechanical (e.g. fans, blowers)<br>☐ Both<br>☐ Do not know
Hygiene facilities in the company (please tick box if apply)	☐ Possibility to wash hands<br>☐ Take shower<br>☐ Separate place for working clothes

WORKER ID............................

	☐ Specific place for the storage of respiratory protective equipment<br>☐ Other (please specify)...<br>☐ ....
Have the work conditions been normal during the work day? (please circle)	Yes No<br>If not normal, please specify (e.g. higher production levels, equipment breakdowns, etc.)

WORKER ID............................

4. Job description: Spray coating of vehicles with di-isocyanate containing paints and primers in the manufacturing or repair of the vehicles (please list the type of work tasks you have been involved in today)

	Work task	Duration of the task in a work shift (hours/ minutes)	Frequency of the task (x times per week)	PPE* used (add the correspon ding numbers)	LEV** used (yes, no)
1	Preparation tasks: decanting, mixing of paints or primers, refilling of apparatus				
2	Spraying in spray cabin/spray booth				
3	Spraying outside of spray booth<br>3.1 Does this involve spraying above the head height (circle): yes no				
4	Surface treatment in automatic spray tunnel				
5	Surface treatment by rolling (small to medium sized areas)				
6	Surface treatment by brushing or pen stick (small areas/touch-up)				
7	Drying/self-curing				
9	Cleaning and maintenance of equipment				
10	Infrequent maintenance activities				
11	Waste management				
12	Other (please specify)				

*PPE (Personal protective equipment) worn:
1. Powered or air-fed, filtering respirator
2. Reusable half or full face mask respirator (not powered or air-fed)
3. Disposable face mask
4. Other Respiratory Protection Equipment (please specify)
5. Coveralls
6. Reusable Gloves
7. Disposable gloves
8. Other (please specify)
** LEV=local exhaust ventilation

If used, has your half or full face mask respirator been fit tested? (please circle)	Yes<br>If yes, when?	No
Have you received	Yes	No

WORKER ID……………………….

information, instruction or training on the use of safe work practices when carrying out this activity? (please circle)	If yes, when?
What kind of general ventilation is used in the workplace when di-isocyanates are being used?	☐ Natural (e.g. open doors / windows)<br>☐ Mechanical (e.g. fans, blowers)<br>☐ Both<br>☐ Do not know
Hygiene facilities in the company (please tick box if apply)	☐ Possibility to wash hands<br>☐ Take shower<br>☐ Separate place for working clothes<br>☐ Specific place for the storage of respiratory protective equipment<br>☐ Other (please specify)………………………………………………………………<br>☐ …………………………………………………………………………………………
Have the work conditions been normal during the work day? (please circle)	Yes No<br>If not normal, please specify (e.g. higher production levels, equipment breakdowns, etc.)

WORKER ID.............................

5. Job description: Welding, grinding or flame cutting of polyurethane-painted materials or e.g. polyurethane-insulated heating pipes (please list the type of work tasks you have been involved in today)

	Work task	Duration of the task in a work shift (hours/ minutes)	Frequency of the task (x times per week)	*PPE* used (add the corresponding numbers)**	*LEV** used (add the corresponding numbers)***
1	Manual welding				
2	Manual tack-welding				
3	Other manual tasks: Grinding or Cutting				
4	Cleaning and maintenance of equipment				
5	Waste management				
6	Other (please specify)				

*PPE (Personal protective equipment) worn:
1. Welding helmet with powered or air-fed, filtering respirator
2. Welding helmet with half mask re-usable dust respirator
3. Welding helmet with disposable particulate respirator
4. Welding helmet without any respirator
5. Welding helmet with other respiratory protection equipment (please specify)
6. Fire/flame resistant clothing
7. Welding gloves
8. Other gloves
9. Other (please specify)

**LEV (Local exhaust ventilation) used:
1. Fixed extractor hood
2. Movable extractor hood
3. Extracted work bench
4. Extracted booth
6. Other (please specify)

WORKER ID............................

If used, has your half or full face mask respirator been fit tested? (please circle)	Yes No<br>If yes, when?
Have you received information, instruction or training on the use of safe work practices when carrying out this activity? (please circle)	Yes No<br><br>If yes, when?
Hygiene facilities in the company (please tick box if apply)	☐ Possibility to wash hands<br>☐ Take shower<br>☐ Separate place for working clothes<br>☐ Specific place for the storage of respiratory protective equipment<br>☐ Other (please specify)..<br>☐ ....
Have the work conditions been normal during the work day? (please circle)	Yes No<br>If not normal, please specify (e.g. higher production levels, equipment breakdowns, etc.)

Operational conditions in welding

Type of the diisocyanate containing insulation material used?	Polyurethane foam Other (please specify)
What welding method was used? (please tick box)	☐ MMA (manual metal arc)<br>☐ Other (please specify)..<br>..
Was the welded material painted? (please circle)	Yes No Don't know
What were the welding voltage and current used?	Voltage: .... V<br>Current: .... A<br><br>Don't know (please circle if apply)
Where do you weld? (please tick box if apply)	☐ Outdoor<br>☐ Outdoor in a constricted area/trench<br>☐ Other (please specify)..<br>..
What kind of general ventilation is used in the workplace?	☐ Natural (e.g. open doors / windows)<br>☐ Mechanical (e.g. fans, blowers)<br>☐ Both<br>☐ Do not know
During welding did you need to	☐ Almost always, i.e. more than 90% arc time

WORKER ID............................

position your head in the fume plume? (please tick box if apply)	☐ Often, i.e. more than half the arc time<br>☐ Sometimes, i.e. less than half arc time<br>☐ Almost never, i.e. <10% of arc time

WORKER ID............................

E. QUESTIONNAIRE ON RESPIRATORY HEALTH AND SYMPTOMS FOR WORKERS (filled also by control workers)

1. Have you had any of these medical problems diagnosed by a doctor?

	yes	no	if yes, insert the year of diagnosis
Chest infection (pneumonia) during the past 12 months			
Sinusitis or any other sinus trouble during the past 12 months			
COPD (chronic bronchitis, chronic pulmonary disease, emphysema)			
Lung cancer			
Asthma			
Hay fever, allergic rhinitis (caused e.g. by animals)			
Other respiratory disease, specify:			
Any heart disease			

2. If you have had asthma, please answer the following:

	yes	no
Have you had asthma attack in the last 12 months?		
Are you currently taking any medicines, including inhalers, aerosols or tablets for asthma?		
Are you taking a rescue inhaler or nebulizer?<br>If yes, can you specify how often?		

3. Cough

	yes	no
Have you been woken with an attack of cough in the last 12 months?		
Do you usually cough when you get up in the morning?		
Do you usually cough at other times during the day?<br>If yes, when?		

If you answered no to all of questions in section 3, go directly to section 4.

	yes	no	
Do you usually bring up any phlegm from the chest if you cough?			

WORKER ID……………………….

	Same	Better	Worse
Is the cough the same, better or worse on days off?			
Is the cough the same, better or worse on holidays?			
How long have you had this cough?	Years<br>Months		

4. Wheezes, whistling and chest tightness

	yes	no
Have you had wheezing or whistling in your chest in the last 12 months?		
Have you woken up with a wheezing or whistling in your chest in the last 12 months?		
Have your chest become tight or breathing difficult in the last 12 months?		
Have you woken up with a feeling of tightness in your chest in the last 12 months?		

If you answered no to all of questions in section 4, go directly to section 5.

	Same	Better	Worse
Is the wheezing, whistling or chest tightness the same, better or worse on days off?			
Is the wheezing, whistling or chest tightness the same, better or worse on holidays?			
If you have had wheezing or whistling, how long have you had it?	Years<br>Months		
If you have had chest tightness, how long have you had it?	Years<br>Months		

5. Shortness of breath

	yes	no
Have you had shortness of breath in the last 12 months?		
Have you woken up with a shortness of breath in your chest in the last 12 months?		
Do you get breathless going up one flight of stairs at your normal pace?		
Do you get breathless when you walk with other people of your own age on the flat at a normal pace?		

If you answered no to all of questions in section 4, go directly to section 5.

WORKER ID............................

	Same	Better	Worse
Is the shortness of breath the same, better or worse on days off?			
Is the shortness of breath the same, better or worse on holidays?			
How long have you had shortness of breath?	Years<br>Months		

6. Eye and nasal symptoms

	yes	no
Have you suffered from eye symptoms such as pricking, itching, burning, dryness, watering, soreness or stinging in the last 12 months?		
Have you suffered from nasal symptoms such as pricking, itching, burning, sneezing or runny, dry or blocked nose in the last 12 months?		

If you answered no to all of questions in section 6, go directly to question 7.

	Same	Better	Worse
Are the eye or nasal symptoms the same, better or worse on days off?			
Are the eye or nasal symptoms the same, better or worse on holidays?			
If you have had nasal or eye symptoms, how long have you had those?	Years<br>Months		
If you have had nasal or eye symptoms, how long have you had those?	Years<br>Months		

7. Have you sought medical attention due to the continuous cough, wheezing, tightness in the chest or shortness of breath (please, circle)?

   Yes / No

   If yes, describe when:

WORKER ID............................

8. COVID-19 Symptoms and diagnosis

Have you had one or more of the following symptoms during the past 14 days? (please tick box if apply)	☐ Cough<br>☐ Sore throat<br>☐ Headache<br>☐ Muscle ache/pain<br>☐ Fever<br>☐ Dyspnoea (difficult breathing)<br>☐ Reduced or loss of senses of taste and smell<br>☐ Nausea or vomiting<br>☐ Diarrhoea<br>☐ Chest pains<br>☐ Skin rashes
Have you been diagnosed with coronavirus disease (COVID-19) based on a test by a doctor/general practitioner/other? (please circle)	Yes No<br><br>If yes, when?
Have you ever been hospitalised due to COVID-19? (please circle)	Yes No<br><br>If yes, when?
Have you been vaccinated past week? (please circle)	Yes No<br><br>If yes, against what?
Have you been vaccinated against severe acute respiratory syndrome coronavirus 2 (SARS-CoV-2)? (please circle)	Yes No<br><br>If yes, please give the date (dd/mm/yyyy)?
